# Associations between sex hormones and obesity-related indicators: results from the NHANES and Mendelian randomization study

**DOI:** 10.1186/s40001-025-03470-x

**Published:** 2025-11-27

**Authors:** Yong Yuan, Shuhao Cui, Yingqi Hou, Xiaofan Meng, Yanqiu Huang, Feng Xu, Wentao Shi

**Affiliations:** 1 Clinical Research Unit, Shanghai Ninth People’s Hospital, Shanghai Jiao Tong University School of Medicine, Shanghai, China; 2 Department of Obstetrics and Gynecology, Shanghai Putuo Central Hospital, Shanghai University of Traditional Chinese Medicine, Shanghai, China; 3 Department of Orthopaedics, Peking University Third Hospital, Beijing, China; 4 Department of Laboratory Medicine, Affiliated Hospital of Changchun University of Chinese Medicine, Changchun, China; 5 School of Public Health, Shanghai Jiao Tong University School of Medicine, Shanghai, China; 6 Shanghai JingAn District ZhaBei Central Hospital, Shanghai, China

**Keywords:** Sex hormones, Obesity-related indicators, National Health and Nutrition Examination Survey, Mendelian randomization, Causation

## Abstract

**Objective:**

This study sought to explore the relationships between sex hormones and obesity-related markers, while utilizing Mendelian randomization (MR) to evaluate their causal links.

**Methods:**

Data from 5179 participants of the 2013–2016 National Health and Nutrition Examination Survey (NHANES) were analyzed. Multivariate linear regression and generalized additive models were utilized to examine linear and non-linear associations between sex hormones and obesity-related parameters. Causal inference was explored via a two-sample MR analysis, with inverse-variance weighting (IVW) as the primary approach.

**Results:**

Cross-sectional assessments showed that in males, testosterone levels, SHBG, and T/E2 ratio were negatively correlated with obesity, while estradiol displayed a non-linear, inverted U-shaped relationship. In females, estradiol and SHBG had negative associations with adiposity measures. MR analysis revealed that genetically increased Bio-T was associated with lower obesity risk in males, while SHBG showed a negative correlation with visceral and waist obesity. In females, Bio-T and FAI were associated with higher visceral adipose tissue volume and waist circumference, findings consistent with potential causal effects.

**Conclusions:**

Sex hormones show sex-specific associations with obesity. Testosterone is protective against obesity in males, whereas female hyperandrogenism elevates the risk of obesity, particularly central obesity. These results offer novel perspectives for obesity-related health interventions.

**Supplementary Information:**

The online version contains supplementary material available at 10.1186/s40001-025-03470-x.

## Introduction

The World Health Organization (WHO) characterizes obesity as excess fat storage, which is closely associated with cardiovascular disease (CVD) risk components such as hypertension, type 2 diabetes (T2D), and dyslipidemia [1, 2]. In 2016, WHO documented a global 39% prevalence among overweight adults and 13% among obese adults [3], emphasizing its substantial public health significance. While body mass index (BMI) is commonly employed as a clinical measure for adiposity, it lacks the ability to reflect fat distribution patterns [1]. Specifically, abdominal obesity (waist circumference > 102 cm in males, > 88 cm in females) and android obesity (truncal fat accumulation, “apple” shape) correlate with elevated CVD risk and mortality from lipid metabolism disorders. Visceral adipose tissue (VAT) actively secretes pro-inflammatory cytokines and adipokines that promote insulin resistance and vascular dysfunction, directly driving CVD pathogenesis [4]. In contrast, gynoid obesity (lower-body fat at hips/thighs, “pear” shape) may reduce susceptibility to T2D and CVD [5]. This protective effect is attributed to subcutaneous fat in the gluteofemoral region, which acts as a metabolic “buffer” by storing excess lipids without inducing systemic inflammation [6]. Even regional fat in extremities (e.g., arm/leg fat) exhibits distinct health implications. For instance, reduced limb fat mass in females has been linked to increased central obesity risk and hyperandrogenism-related metabolic disturbances [7], while preserved leg fat in older adults correlates with lower T2D incidence [8]. Low-quality diets (high-fat diet, sugary drinks, etc.) and drug abuse are often the triggers of obesity [9, 10]. Fat accumulation in different parts of the body may have different cardiovascular outcomes, while increasing physical activity and regulating hormone secretion can effectively improve fat distribution, for example, steroid hormones may change central obesity [11].

Research indicates that sex hormones play a crucial role in regulating fat mass and its distribution during puberty in both genders [5]. In women, estrogen works in synergy with adipose tissue genes to enhance the mass of gluteofemoral subcutaneous fat in those of reproductive age, while decreasing central adipose tissue mass, thus alleviating the load of cardiovascular disease (CVD) risk factors [8]. Nevertheless, elevated estrogen levels in women are linked to a higher risk of uterine fibroids or reproductive malignancies [12]. Multiple research studies suggest that hyperandrogenism in females is closely related to polycystic ovary syndrome, frequently accompanied by heightened intra-abdominal fat accumulation [13]. The free androgen index (FAI) serves as a proxy for free testosterone in women and is widely utilized in investigations of hyperandrogenism [14]. Conversely, males with diminished testosterone levels (evidenced by increased upper body fat and visceral adipose tissue mass) [5] face a notably higher risk of type 2 diabetes (T2D) and cardiovascular disease (CVD). Additionally, their overall mortality rate is markedly higher compared to women [15]. During the middle to late stages of puberty, the testes and peripheral tissues might generate relatively more estrogen before testosterone production reaches adult levels, contributing to the development of gynecomastia [16]. Presently, the testosterone-to-estradiol (T/E2) ratio is being established as a biomarker for a balanced hormonal environment [17] and has been identified as a predictor of cardiovascular disease and overall mortality [18]. Furthermore, sex hormone-binding globulin (SHBG) is a factor that modulates the biological effects of steroid hormones [19] and is frequently linked to metabolic abnormalities [17]. Genetic disruption of hypothalamic–pituitary–gonadal signaling underscores the causal link between gonadotropin defects and androgen deficiency. For example, CCDC141 variants are implicated in congenital hypogonadotropic hypogonadism, highlighting how germline architecture can shape circulating sex steroids and downstream metabolic risk [20].

Research has shown that sex hormones play a pivotal role in regulating body fat distribution, giving rise to diverse obesity phenotypes. However, existing observational studies are hampered by small, unrepresentative sample sizes and insufficient consideration of fat distribution patterns and obesity classifications. In light of these limitations, Mendelian randomization (MR) studies, which use genetic data as an instrumental variable to clarify phenotype relationships, offer a valuable supplementary approach to explore causal links. Since genetic alleles are randomly assigned during meiosis and independent of environmental factors, MR-derived genetic associations are less susceptible to confounding bias or reverse causation risks [21]. Notably, some MR studies have only focused on causal connections between sex hormones, BMI, and waist-to-hip ratio (WHR), neglecting crucial indicators such as arm circumference and regional fat masses in the arms, legs, and hips [5, 22]. Recognizing the distinct health implications of pear-shaped (dominated by lower-body fat) and apple-shaped (dominated by upper-body fat) body types, incorporating site-specific fat content is vital for a comprehensive analysis.

Therefore, this study aimed to investigate the association between sex hormones and obesity-related markers. By leveraging data from the National Health and Nutrition Examination Survey (NHANES) and applying MR methods in a public genetic database, the research explored causal relationships, addressing the gaps in previous studies and providing a more nuanced understanding of the hormonal influences on obesity. Beyond endocrine regulation, environmental and thermal stressors can influence testicular function and androgen biosynthesis. Heat stress perturbs spermatogenesis and testicular homeostasis through oxidative stress, ER-stress, and apoptosis-related pathways, potentially lowering circulating testosterone and altering downstream metabolic risk [23].

## Methods

### Overall study design

The present study comprised two sequential phases, as illustrated in Figure S1. In the first phase, we leveraged participant data from the NHANES database to conduct generalized additive modeling and linear regression analyses, aiming to explore the relationships between steroid hormones and obesity-associated markers. The second phase involved MR analyses on aggregated Genome-Wide Association Study (GWAS) datasets. This approach was used to evaluate causal links between heritably determined steroid hormone levels and obesity-related indicators, thereby addressing the causal inference gap in observational research.

### Data sources and study population

This study employed two cycles of publicly available NHANES data from 2013 to 2016. Males and premenopausal females (hereafter “females”) were included, with menopausal status evaluated via a reproductive health questionnaire. Specifically, participants were asked, “Have you had at least one menstrual period in the last 12 months?” Those responding “No” were further asked: “What is the reason you have not had a period in the last 12 months? (Options: pregnancy, breastfeeding, hysterectomy, menopause/life change, other)”. Females with no menstrual period in the past 12 months were classified as postmenopausal, and those with a history of sex hormone use were excluded to mitigate bias [19]. After excluding postmenopausal females, participants lacking physical examinations (body measures, X-ray), sex hormone data, or covariates, a total of 5179 subjects were included. All analyses used NHANES public data, approved by the National Center for Health Statistics (NCHS) Research Ethics Review Board, with written informed consent from all participants. A flowchart detailing participant selection is shown in Figure S2.

### Measurement of sex hormones

The isotope dilution liquid chromatography–tandem mass spectrometry, developed by the National Center for Disease Control and Prevention (CDC), enables direct quantification of total testosterone and estradiol. MEC operates 5 days/week (with rotating non-business days). Participants were randomly assigned to morning, afternoon, or evening sessions. Participants aged ≥ 12 in morning sessions were required to fast for 9 h; fasting status was verified by phlebotomists before blood collection. Blood samples were processed, aliquoted into vials, refrigerated or frozen at MEC, then shipped to laboratories across the U.S. The lower limit of detection (LLOD) for total testosterone (ng/dL) is 0.75 ng/mL, and for estradiol (pg/mL) is 2.994 pg/mL. For SHBG assessment, the method leverages the binding interaction between SHBG and an immunizing antibody, with chemiluminescent detection of resultant reaction products. The T/E2 ratio is calculated as $0.1\times total testosterone (ng/dL) / estradiol (pg/mL)$[14]. FAI is calculated using the following formula: $100\times total testosterone (nmol/L)/ SHBG (nmol/L)$[24].

### Measurement of obesity-related indicators

Trained health technicians at the Mobile Examination Center (MEC) obtained anthropometric data (BMI, waist circumference, arm circumference, sagittal mean abdominal diameter) through standardized measurements. Whole-body dual-energy X-ray absorptiometry (DXA) scans were conducted for all participants, quantifying fat distribution in android/gynoid regions (including trunk, visceral adipose tissue [VAT], and subcutaneous adipose tissue [SAT]). The Hologic APEX software, utilized in scan analysis, defined regions for VAT, SAT, and the android-to-gynoid (A/G) ratio.

### Assessment of covariates

Demographic information (age, sex, ethnicity, education, household income) and lifestyle elements (smoking, alcohol consumption, diet, physical activity) were collected through questionnaire interviews. Non-smokers were defined as individuals with a lifetime smoking history of less than 100 cigarettes, while all others were designated as smokers [25]. In the analyses, alcohol consumption was handled as a continuous variable. Dietary intake was assessed using two 24-h dietary surveys, with overall diet quality measured by the healthy eating index 2015 (HEI—2015), which produces scores from 0 to 100 (higher scores indicate healthier diets). Total leisure-time physical activity (LTPA) was calculated as the sum of moderate-intensity recreational activity minutes and double the duration of high-intensity recreational activity. Based on the 2018 Physical Activity Guidelines for Americans, participants were divided into groups: inactive (with no LTPA), underactive (where 0 < LTPA < 150 min per week), or sufficiently active (with LTPA ≥ 150 min per week) [26].

### Statistical analysis

Data across two survey cycles were integrated with 4-year sampling weights to maintain representativeness. Group comparisons utilized Rao–Scott Chi-square tests and *t*-tests. Generalized additive models were applied to characterize smooth associations between continuous hormone metrics and obesity markers, accounting for covariates. Sex hormones, stratified by tertiles, underwent multivariate linear regression to assess their impacts on obesity, with β-coefficients and 95% confidence intervals computed. Model 1 controlled for age, sex, and race; Model 2 additionally incorporated education, income, smoking, alcohol intake, physical activity, and dietary patterns. Analyses were performed using SAS 9.4.7 and R 4.2.3, with a two-tailed significance criterion of 0.05.

### Mendelian randomization

#### Basic concept of MR analysis

In MR analysis, valid instrumental variables (IVs) need to follow the following criteria: (1) single-nucleotide polymorphisms (SNPs) are strongly associated with sex hormones; (2) SNPs are not associated with confounders; and (3) SNPs affect obesity-related indicators only through sex hormones (Figure S3).

#### Data sources

For two-sample MR, we selected 4 sex hormone indicators (FAI, Bio-T, estradiol, SHBG, stratified by sex) and 16 obesity-related indicators. Genetic data for FAI were retrieved from a meta-analysis of UKB and FinnGen [27]. Bio-T, estradiol, and SHBG genetic data were all obtained from relevant UKB studies [28, 29]. Obesity-related genetic datasets were sourced from GIANT, UKB, HISLA, and consortia of European/African ancestries [30–33]. Included studies were approved by their institutional review boards (IRBs) and ethics committees. Detailed information is provided in Table S1.

#### Selection of SNPs for MR analysis

SNPs with genome-wide significant associations to sex hormones (*P* < 5 × 10^–8^) underwent initial screening. Subsequently, SNPs within the target gene region and its adjacent > 10,000 kb window were extracted, with linkage disequilibrium (LD) clustering applied in the European 1000 Genome dataset (*r*^2^ < 0.001). Moreover, SNPs showing genome-wide significance for obesity indicators (*P* < 1.0 × 10^–5^) were excluded. The F-statistic was calculated to remove SNPs with *F* < 10.

#### MR and sensitivity analysis

Inverse variance weighting (IVW) served as the core analytical technique to examine causal links between sex hormones and obesity-related indicators under a random-effects framework. MR-Egger and weighted median methods were employed for result validation. Our two-sample MR workflow (harmonization, clumping, IVW as primary, with weighted median and MR-Egger sensitivity) follows current practice applied across complex traits, including recent analyses of circulating immune cell traits and cancer risk [34]. Heterogeneity was gauged using Cochran’s Q test. Horizontal pleiotropy was identified via MR-Egger regression (intercept indicating directional pleiotropy) and funnel plot symmetry assessment. Leave-one-out sensitivity analysis was utilized to verify result stability. Multidimensional pleiotropy was scrutinized through the MR-PRESSO test. All procedures were executed with R 4.2.3 and the TwoSampleMR software package.

## Results

### Population characteristics of NHANES

A total of 5179 individuals were finally included in the analysis during the two cycles 2013–2016, of which 2870 (55.4%) were males and 2309 (44.6%) were females, whose main characteristics are shown in Table 1. Compared to males, females were younger, consumed less alcohol, and had lower smoking prevalence, but had higher HEI scores. These two groups differed significantly in racial composition, household poverty ratio, and marital status, while education and history of diabetes did not differ significantly. There were significant differences between the two groups in the comparison of 16 obesity-related indicators and sex hormone levels.

**Table 1:** Baseline characteristics: NHANES (2013–2016)

Characteristics	Total (*N* = 5179)	Male (*N* = 2870)	Female (*N* = 2309)	*P*-value
Demographic information				
Age, years, mean (SE)	31.6 (0.2)	32.5 (0.3)	30.5 (0.3)	< 0.001
Alcohol consumption, gram, mean(SE)	24.7 (0.5)	30.8 (0.7)	17.1 (0.5)	< 0.001
HEI-2015, mean (SE)	48.5 (0.2)	47.7 (0.3)	49.5 (0.3)	< 0.001
Ethnicity, *n* (%)				
Mexican American	1041 (20.1)	543 (18.9)	498 (21.6)	0.005
Other Hispanic	588 (11.4)	298 (10.4)	290 (12.6)	
Non-Hispanic White	1711 (33.0)	992 (34.6)	719 (31.1)	
Non-Hispanic Black	986 (19.0)	558 (19.4)	428 (18.5)	
Other race—including multi-racial	853 (16.5)	479 (16.7)	374 (16.2)	
Education levels, *n* (%)				
Less than high s chool	1936 (37.4)	1064 (37.1)	872 (37.8)	0.609
High school or above	3243 (62.6)	1806 (63.0)	1437 (62.2)	
Marriage status, *n* (%)				
Have a partner or be married	2277 (44.0)	1335 (46.5)	942 (40.8)	< 0.001
Single or widowed	2902 (56.0)	1535 (53.5)	1367 (59.2)	
Poverty status, *n* (%)				
≤ 1.30	1989 (39.2)	1048 (37.2)	941 (41.7)	0.002
1.30–3.50	764 (15.1)	423 (15.0)	341 (15.1)	
> 3.50	2324 (45.8)	1347 (47.8)	977 (43.3)	
Recent tobacco use, *n* (%)				
Yes	1124 (21.7)	745 (26.0)	379 (16.4)	< 0.001
No	4055 (78.3)	2125 (74.0)	1930 (83.6)	
Leisure time physical activity, *n* (%)				
0	2009 (38.8)	1049 (36.6)	960 (41.6)	< 0.001
0–150	779 (15.0)	361 (12.6)	418 (18.1)	
≥ 150	2391 (46.2)	1460 (50.9)	931 (40.3)	
History of diabetes, *n* (%)				
Yes	251 (4.9)	149 (5.2)	102 (4.4)	0.197
No	4928 (95.2)	2721 (94.8)	2207 (95.6)	
Sex hormones and indicators of obesity and adiposity				
Sex hormones and related indices				
Total testosterone, ng/dL, mean(SE)	248.5 (3.4)	427.3 (3.5)	26.2 (0.4)	< 0.001
Estradiol, pg/mL, mean(SE)	52.1 (0.9)	23.8 (0.2)	87.4 (1.7)	< 0.001
SHBG, nmol/L, mean (SE)	50.0 (0.5)	36.9 (0.4)	66.3 (1.0)	< 0.001
T/E2 ratio, mean (SE)	–	1.9 (0.02)	–	< 0.001
FAI, mean (SE)	–	–	1.9 (0.04)	–
Body measurements				
Body mass index, kg/m^2^, mean(SE)	27.7 (0.1)	27.3 (0.1)	28.1 (0.2)	< 0.001
Waist circumference, cm, mean(SE)	93.5 (0.2)	94.4 (0.3)	92.3 (0.4)	< 0.001
Arm circumference, cm, mean(SE)	32.3 (0.1)	33.2 (0.1)	31.2 (0.1)	< 0.001
Average sagittal abdominal diameter, cm, mean(SE)	21.2 (0.1)	21.6 (0.1)	20.8 (0.1)	< 0.001
Android/gynoid measurements				
Android fat mass, gram, mean(SE)	2145.4 (18.2)	2066.5 (24.6)	2243.4 (26.9)	< 0.001
Gynoid fat mass, gram, mean(SE)	4291.3 (26.1)	3696.5 (30.7)	5030.6 (39.5)	< 0.001
Subcutaneous fat mass, gram, mean(SE)	1468.3 (11.5)	1178.4 (13.7)	1828.7 (16.7)	< 0.001
Total abdominal fat mass, gram, mean(SE)	1896.7 (14.0)	1630.6 (17.4)	2227.5 (20.9)	< 0.001
Visceral adipose tissue mass, gram, mean(SE)	428.4 (3.7)	452.2 (4.9)	398.8 (5.5)	< 0.001
Whole body measurements				
Head fat, gram, Mean (SE)	1166.6 (2.3)	1238.2 (2.9)	1077.7 (2.7)	< 0.001
Left arm fat, gram, mean(SE)	1559.1 (11.2)	1369.9 (13.3)	1794.4 (17.6)	< 0.001
Left leg fat, gram, mean(SE)	4570.4 (29.2)	3902.2 (33.7)	5401.0 (44.8)	< 0.001
Right arm fat, gram, mean(SE)	1549.5 (11.2)	720.5 (13.4)	1782.8 (17.7)	< 0.001
Right leg fat, gram, mean(SE)	4669.0 (29.84)	3979.0 (34.3)	5526.8 (45.7)	< 0.001
Trunk fat, gram, mean (SE)	11,834.5 (87.7)	10,966.0 (113.7)	12,914.0 (133.3)	< 0.001
Total fat, gram, mean (SE)	25,349.3 (162.1)	22,817.1 (201.3)	28,496.6 (248.6)	< 0.001

Descriptive data were shown as mean (SE) while categorical variables were reported as *n* (%). *P*-values less than 0.05 (*P*-value < 0.05) were considered significant. *SE* standard error of mean, *HEI* healthy eating index, *CVD* cardiovascular disease, *N* number, *SHBG* sex hormone-binding globulin, *T/E2* testosterone-to-estradiol, *FAI* free androgen index

### Associations of sex hormones, SHBG, or T/E2 ratio with obesity-related indicators in males

**Fig. 1:**
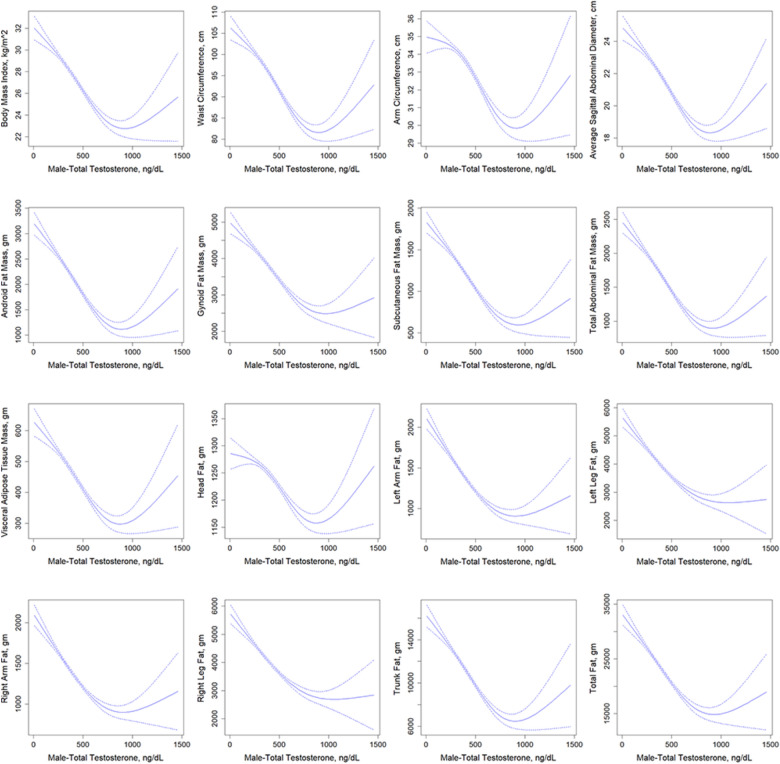
Smoothed curves derived from a generalized additive model illustrating the relationship between total testosterone (continuous variable) and 16 obesity-related indicators (continuous variable). Model was adjusted for age, ethnicity, education level, poverty status, smoking status, HEI-2015, alcohol consumption and leisure-time physical activity. *gm* gram

Using tertile groupings of total testosterone, estradiol, and SHBG, we examined relationships with 16 obesity-associated markers in male subjects (Table 2, Table S2, Table S3). Smoothed curves (solid line) and 95% confidence intervals (dashed line) for associations between these hormones, the T/E2 ratio, and the 16 indicators—adjusted for covariates—are presented in Fig. 1, Figure S4–S6. Total testosterone, SHBG, and the T/E2 ratio exhibited negative correlations with all 16 obesity-related metrics (*P* < 0.05). Specifically, SHBG showed significant negative associations with 14 indicators across three tertiles in both models (*P* < 0.05), with only non-significant links to visceral and head adiposity in the second tertile (*P* > 0.05). In contrast, total testosterone demonstrated consistent significant negative relationships with all 16 indicators in the upper two tertiles (*P* < 0.05). For estradiol, smoothed curves for 14 indicators (excluding visceral and head fat mass) revealed an inverted U-shaped relationship. At a mean estradiol level of 23.8 pg/mL in males, these 14 indicators showed significant positive correlations with estradiol (*P* < 0.05).

**Table 2:** Association of total testosterone with 16 obesity-related indicators in males from NHANES 2013–2016 (*n* = 2870)

		Male—total testosterone, ng/dL					
		Q1 (< 336.0 ng/dL)		Q2 (336.0–480.0 ng/dL)		Q3 (≥ 480.0 ng/dL)	
		*β* (95% CI)	*P*-value	*β* (95% CI)	*P*-value	*β* (95% CI)	*P*-value
Body measures							
Body mass index, kg/m^2^	Model 1	−0.009 (−0.017, −0.0002)	0.046	−0.016 (−0.028,−0.005)	0.007	−0.007 (−0.010,−0.004)	< 0.001
	Model 2	−0.013 (−0.021, −0.004)	0.005	−0.017 (−0.027,−0.006)	0.003	−0.007 (−0.010,−0.004)	< 0.001
Waist circumference, cm	Model 1	−0.020 (−0.0410, 0.000)	0.054	−0.047 (−0.076,−0.018)	0.002	−0.020 (−0.029,−0.011)	< 0.001
	Model 2	−0.030 (−0.051,−0.009)	0.006	−0.048 (−0.074,−0.022)	< 0.001	−0.020 (−0.029,−0.010)	< 0.001
Arm circumference, cm	Model 1	−0.001 (−0.007,0.006)	0.834	−0.009 (−0.018,0.001)	0.080	−0.006 (−0.008,−0.003)	< 0.001
	Model 2	−0.005 (−0.011,0.001)	0.099	−0.009 (−0.018,−0.000)	0.049	−0.006 (−0.008,−0.003)	< 0.001
Average sagittal abdominal diameter, cm	Model 1	−0.007 (−0.013,−0.002)	0.014	−0.014 (−0.022,−0.005)	0.002	−0.005 (−0.008,−0.002)	< 0.001
	Model 2	−0.010 (−0.015,−0.004)	< 0.001	−0.014 (−0.021,−0.006)	< 0.001	−0.005 (−0.008,−0.002)	0.001
Android/gynoid measurements							
Android fat mass, gram	Model 1	−2.446 (−4.257,−0.634)	0.010	−4.769 (−7.101,−2.437)	< 0.001	−1.396 (−2.088,−0.703)	< 0.001
	Model 2	−3.202 (−5.108,−1.296)	0.002	−4.855 (−6.944,−2.766)	< 0.001	−1.280 (−2.010,−0.549)	0.001
Gynoid fat mass, gram	Model 1	−1.705 (−4.205,0.794)	0.174	−4.536 (−7.302,−1.771)	0.002	−2.065 (−2.907,−1.224)	< 0.001
	Model 2	−2.854 (−5.329,−0.379)	0.025	−4.683 (−7.179,−2.187)	< 0.001	−1.956 (−2.808,−1.105)	< 0.001
Subcutaneous fat mass, gram	Model 1	−1.017 (−2.012,−0.021)	0.046	−2.678 (−3.962,−1.394)	< 0.001	−0.891 (−1.280,−0.502)	< 0.001
	Model 2	−1.470 (−2.467,−0.473	0.005	−2.725 (−3.856,−1.594)	< 0.001	−0.830 (−1.234,−0.425)	< 0.001
Total abdominal fat mass, gram	Model 1	−1.699 (−2.895,−0.504)	0.007	−3.379 (−5.063,−1.696)	< 0.001	−1.143 (−1.646,−0.639)	< 0.001
	Model 2	−2.165 (−3.380,−0.949)	0.001	−3.432 (−4.912,−1.952)	< 0.001	−1.058 (−1.585,−0.531)	< 0.001
Visceral adipose tissue mass, gram	Model 1	−0.683 (−0.949,−0.416)	< 0.001	−0.701 (−1.208,−0.195)	0.008	−0.252 (−0.383,−0.120)	< 0.001
	Model 2	−0.695 (−0.972,−0.418)	< 0.001	−0.707 (−1.179,−0.234)	0.005	−0.228 (−0.367,−0.089)	0.002
Whole body measurements							
Head fat, gram	Model 1	0.042 (−0.230,0.315)	0.752	−0.349 (−0.621,−0.077)	0.014	−0.157 (−0.263,−0.052)	0.005
	Model 2	−0.062 (−0.326,0.203)	0.637	−0.350 (−0.619,−0.081)	0.013	−0.142 (−0.247,−0.036)	0.010
Left arm fat, gram	Model 1	−1.555 (−2.653,−0.457)	0.007	−1.952 (−3.282,−0.621)	0.006	−0.706 (−1.036,−0.375)	< 0.001
	Model 2	−1.994 (−3.151,−0.837)	0.001	−1.997 (−3.231,−0.764)	0.003	−0.669 (−1.007,−0.331)	< 0.001
Left leg fat, gram	Model 1	−3.300 (−6.111,−0.489)	0.023	−4.444 (−7.566,−1.321)	0.007	−2.180 (−2.942,−1.417)	< 0.001
	Model 2	−4.272 (−7.065,−1.478)	0.004	−4.574 (−7.553,−1.594)	0.004	−2.064 (−2.819,−1.308)	< 0.001
Right arm fat, gram	Model 1	−1.700 (−2.790,−0.609)	0.003	−2.305 (−3.467,−1.142)	< 0.001	−0.748 (−1.080,−0.417)	< 0.001
	Model 2	−2.140 (−3.289,−0.991)	< 0.001	−2.362 (−3.496,−1.227)	< 0.001	−0.703 (−1.043,−0.364)	< 0.001
Right leg fat, gram	Model 1	−3.229 (−5.962,−0.496)	0.022	−4.638 (−7.717,−1.566)	0.004	−2.222 (−2.970,−1.475)	< 0.001
	Model 2	−4.186 (−6.925,−1.446)	0.004	−4.756 (−7.687,−1.825)	0.002	−2.096 (−2.836,−1.355)	< 0.001
Trunk fat, gram	Model 1	−10.462 (−19.686,−1.238)	0.028	−21.927 (−32.526,−11.327)	< 0.001	−6.896 (−10.192,−3.599)	< 0.001
	Model 2	−14.106 (−23.310,−4.902)	0.004	−22.382 (−31.912,−12.852)	< 0.001	−6.310 (−9.753,−2.868)	< 0.001
Total fat, gram	Model 1	−20.203 (−36.704,−3.702)	0.018	−35.614 (−54.092,−17.137)	< 0.001	−12.909 (−18.354,−7.464)	< 0.001
	Model 2	−26.760 (−43.311,−10.209)	0.003	−36.421 (−53.389,−19.453)	< 0.001	−11.983 (−17.577,−6.390)	< 0.001

Model 1 was adjusted for age and ethnicity. Model 2 was adjusted for age, ethnicity, education level, poverty status, smoking status, HEI-2015, alcohol consumption and leisure-time physical activity. *CI* confidence interval, *P*-values less than 0.05 (*P*-value < 0.05) were considered significant

### Associations of sex hormones, SHBG, or FAI with obesity-related indicators in females

**Fig. 2:**
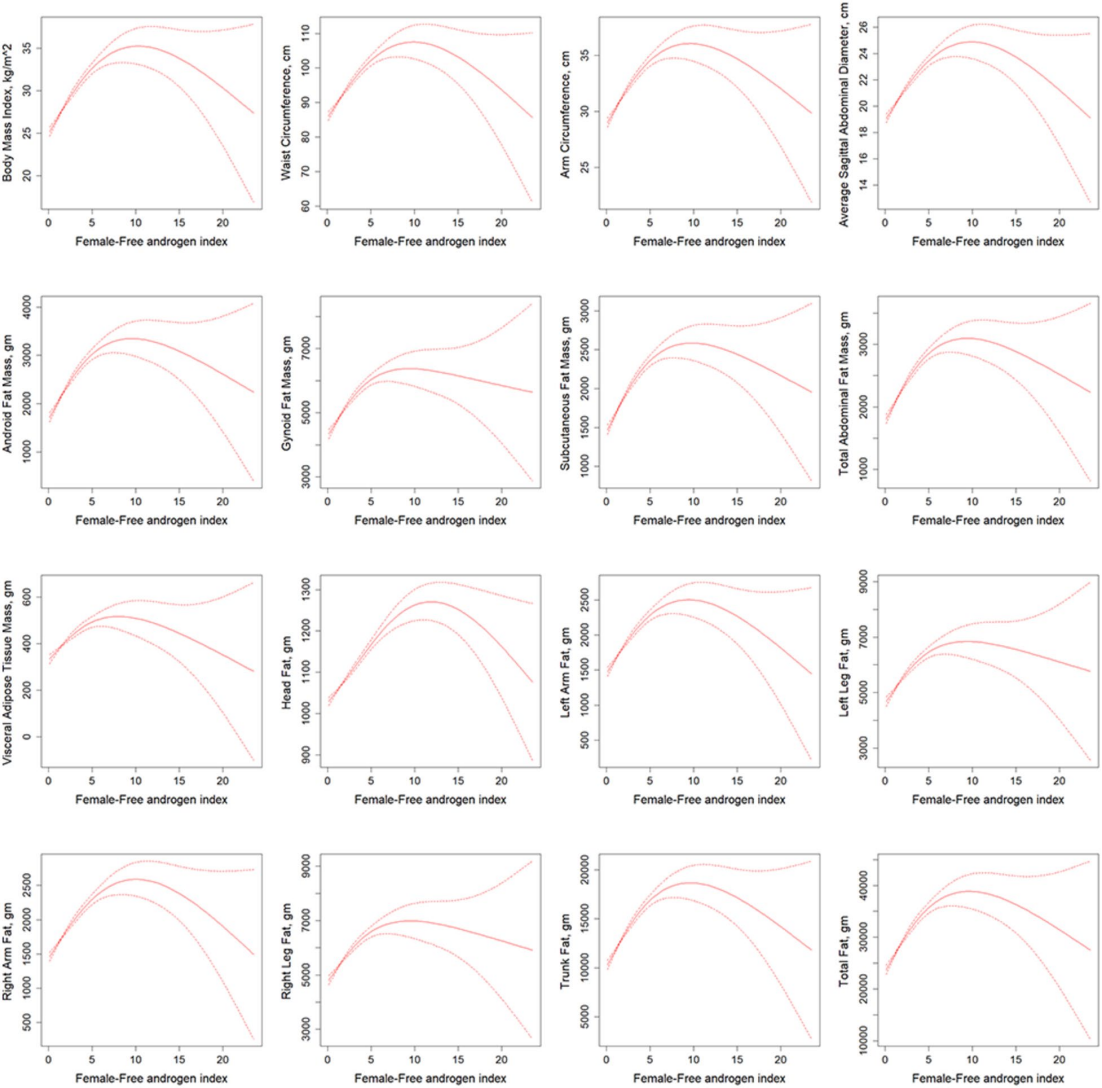
Smoothed curves derived from a generalized additive model illustrating the relationship between free androgen index (continuous variable) and 16 obesity-related indicators (continuous variable) in women. Model was adjusted for age, ethnicity, education level, poverty status, smoking status, HEI-2015, alcohol consumption and leisure-time physical activity. *gm* gram

Compared to males, in addition to total testosterone, estradiol, and SHBG (Figure S7, S8, S9), we additionally added smoothed curves between FAI and 16 indicators (Fig. 2). Estradiol was consistently and significantly negatively correlated with the 16 indicators in the higher two tertiles (*P* < 0.05) (Table 3), which is consistent with the trend of the smoothed curves. In contrast, there was no significant association between total testosterone levels and whole-body adiposity measures and body measurements (*P* > 0.05), and the trends were inconsistent across tertile levels. In addition, inverted U-shaped associations of both total testosterone and FAI with obesity-related indicators were observed, which were consistent with the results of the two models. SHBG showed an inverse trend in the smoothed curves with the obesity-related indicators, and was significantly negatively correlated with 16 indicators at the 1 st, and 2nd tertile levels in both models (*P* < 0.05).

**Table 3:** Association of estradiol with 16 obesity-related indicators in females from NHANES 2013–2016 (*n* = 2309)

		Female—estradiol, pg/mL					
		Q1 (< 37.0 pg/mL)		Q2 (37.0 −98.0 pg/mL)		Q3 (≥ 98.0 pg/mL)	
		*β* (95%CI)	*P*-value	*β* (95%CI)	*P*-value	*β* (95%CI)	*P*-value
Body measures							
Body mass index, kg/m^2^	Model 1	0.114 (0.059,0.169)	< 0.001	−0.055 (−0.079,−0.030)	< 0.001	−0.010 (−0.017,−0.003)	0.005
	Model 2	0.112 (0.057,0.167)	< 0.001	−0.054 (−0.079,−0.028)	< 0.001	−0.010 (−0.018,−0.003)	0.006
Waist circumference, cm	Model 1	0.234 (0.097,0.370)	0.002	−0.108 (−0.172,−0.044)	0.002	−0.022 (−0.040,−0.003)	0.027
	Model 2	0.228 (0.098,0.359)	0.001	−0.102 (−0.167,−0.038)	0.003	−0.021 (−0.004,−0.003)	0.026
Arm circumference, cm	Model 1	0.093 (0.057,0.130)	< 0.001	−0.037 (−0.056,−0.018)	< 0.001	−0.008 (−0.014,−0.003)	0.003
	Model 2	0.093 (0.057,0.129)	< 0.001	−0.036 (−0.055,−0.016)	0.001	−0.008 (−0.014,−0.003)	0.003
Average sagittal abdominal diameter, cm	Model 1	0.049 (0.013,0.085)	0.009	−0.030 (−0.046,−0.014)	< 0.001	−0.007 (−0.011,−0.003)	0.001
	Model 2	0.047 (0.011,0.082)	0.011	−0.029 (−0.045,−0.012)	0.002	−0.007 (−0.011,−0.003)	0.002
Android/gynoid measurements							
Android fat mass, gram	Model 1	17.013 (4.765,29.262)	0.008	−10.318 (−16.318,−4.319)	0.001	−1.944 (−3.358,−0.531)	0.009
	Model 2	16.693 (4.577,28.809)	0.009	−10.001 (−16.272,−3.729)	0.003	−1.923 (−3.302,−0.545)	0.008
Gynoid fat mass, gram	Model 1	23.618 (9.541,37.695)	0.002	−10.788 (−19.403,−2.172)	0.016	−2.709 (−4.714,−0.703)	0.010
	Model 2	23.537 (8.938,38.135)	0.003	−11.055 (−20.437,−1.674)	0.023	−2.674 (−4.772,−0.575)	0.014
Subcutaneous fat mass, gram	Model 1	12.093 (5.133,19.052)	0.001	−6.614 (−9.875,−3.354)	< 0.001	−1.248 (−2.166,−0.329)	0.009
	Model 2	11.948 (5.005,18.892)	0.001	−6.576 (−10.027,−3.124)	< 0.001	−1.236 (−2.156,−0.316)	0.010
Total abdominal fat mass, gram	Model 1	13.030 (4.154,21.906)	0.005	−8.020 (−12.118,−3.922)	< 0.001	−1.497 (−2.696,−0.298)	0.016
	Model 2	12.686 (3.873,21.499)	0.006	−7.922 (−12.201,−3.645)	< 0.001	−1.469 (−2.646,−0.293)	0.016
Visceral adipose tissue mass, gram	Model 1	0.937 (−1.523,3.398)	0.443	−1.406 (−2.530,−0.282)	0.016	−0.249 (−0.563,0.065)	0.116
	Model 2	0.738 (−1.680,3.156)	0.538	−1.347 (−2.521,−0.173)	0.026	−0.234 (−0.526,0.058)	0.113
Whole body measurements							
Head fat, gram	Model 1	1.681 (0.623,2.734)	0.003	−0.455 (−0.869,−0.041)	0.032	−0.086 (−0.235,0.063)	0.247
	Model 2	1.675 (0.631,2.718)	0.003	−0.436 (−0.837,−0.034)	0.034	−0.086 (−0.236,0.063)	0.248
Left arm fat, gram	Model 1	9.735 (3.395,16.076)	0.004	−6.056 (−9.341,−2.779)	< 0.001	−1.216 (−2.038,−0.395)	0.005
	Model 2	9.653 (3.187,16.119)	0.005	−5.984 (−9.448,−2.520)	0.001	−1.207 (−2.050,−0.365)	0.007
Left leg fat, gram	Model 1	28.436 (12.726,44.146)	< 0.001	−11.623 (−21.829,−1.418)	0.027	−3.045 (−5.356,−0.733)	0.012
	Model 2	28.144 (11.683,44.605)	0..002	−12.027 (−22.969,−1.085)	0.032	−3.052 (−5.535,−0.569)	0.018
Right arm fat, gram	Model 1	9.981 (3.512,16.451)	0.004	−6.190 (−9.590,−2.790)	< 0.001	−1.036 (−2.006,−0.067)	0.037
	Model 2	9.945 (3.517,16.373)	0.004	−6.150 (−9.771,−2.529)	0.002	−1.020 (−1.994,−0.045)	0.041
Right leg fat, gram	Model 1	26.848 (10.414,43.282)	0.002	−11.358 (−21.650,−1.067)	0.032	−3.255 (−5.521,−0.990)	0.006
	Model 2	26.652 (9.535,43.769)	0.003	−11.914 (−23.036,−0.793)	0.037	−3.241 (−5.633,−0.848)	0.010
Trunk fat, gram	Model 1	84.536 (25.935,143.138)	0.006	−49.928 (−75.771,−24.086)	< 0.001	−9.032 (−15.969,−2.096)	0.012
	Model 2	83.035 (25.356,140.715)	0.006	−49.344 (−76.466,−22.222)	< 0.001	−8.890 (−15.740,−2.041)	0.013
Total fat, gram	Model 1	161.218 (72.813,249.623)	< 0.001	−85.615 (−135.281,−35.949)	0.001	−17.671 (−30.405,−4.937)	0.008
	Model 2	159.104 (69.491,248.717)	0.001	−85.855 (−138.681,−33.029)	0.002	−17.496 (−30.505,−4.488)	0.010

Model 1 was adjusted for age and ethnicity. Model 2 was adjusted for age, ethnicity, education level, poverty status, smoking status, HEI-2015, alcohol consumption and leisure-time physical activity. *CI* confidence interval, *P*-values less than 0.05 (*p* < 0.05) were considered significant

### MR of sex hormones and obesity-related indicators in males

**Fig. 3:**
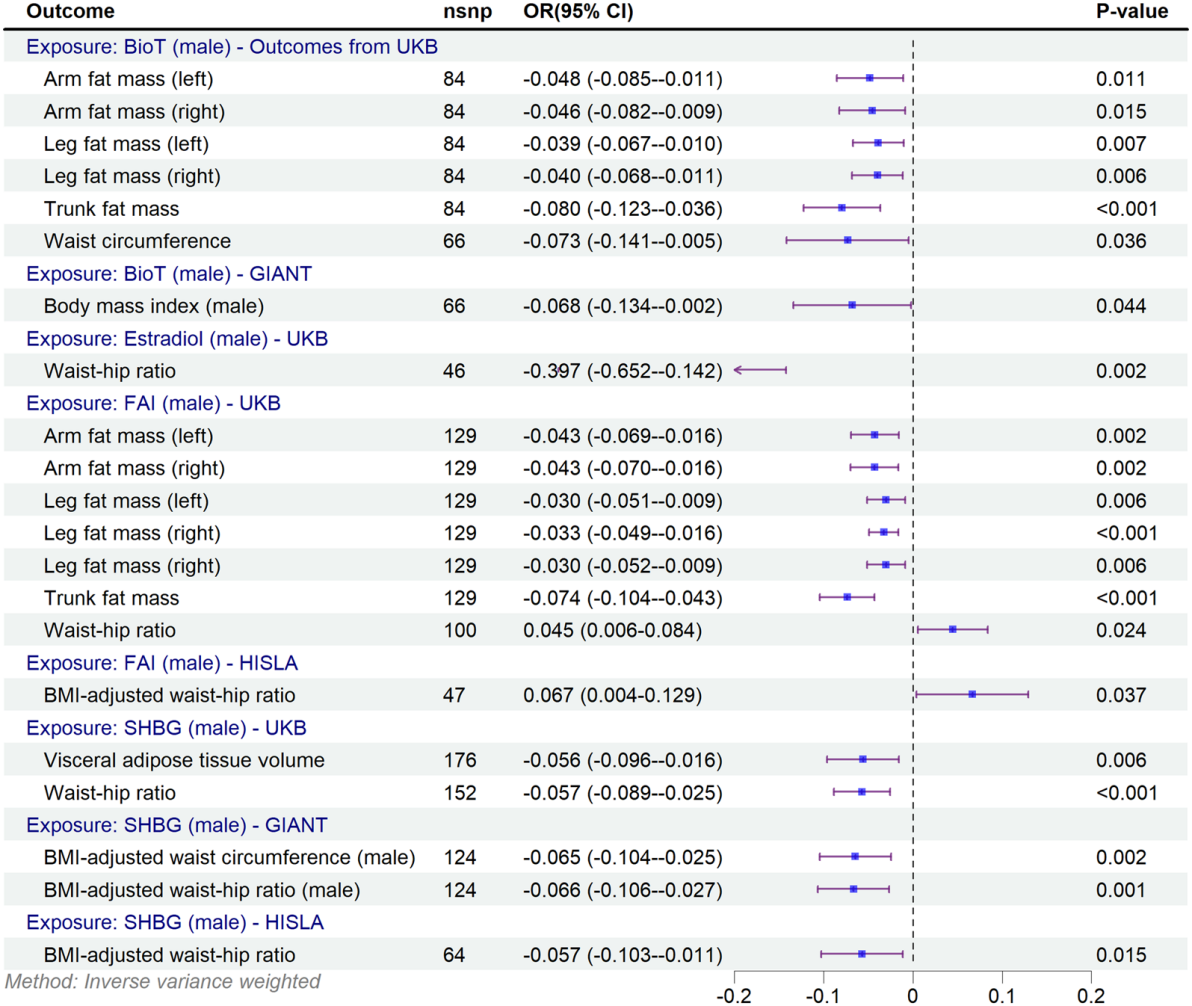
Forest plot of the Mendelian randomization study investigating the effect of sex hormones on obesity-related indicators in males. *BioT* bioavailable testosterone, *BMI* body mass index, F*AI* free androgen index, SHBG sex hormone-binding globulin. *P*-values less than 0.05 (*p* < 0.05) were considered significant

IVW analyses using a random-effects model revealed associations between sex hormones and obesity indicators in males, consistent with significant potential causal effects (Fig. 3). A higher FAI for gene compensation was associated with reduced total body adiposity but was associated with increased WHR [β (95%CI) 0.045 (0.006, 0.084)] and BMI-adjusted WHR [0.067 (0.004, 0.129)]. Bio-T was also associated with decreased total body adiposity, and showed an inverse association with waist circumference—findings consistent with potential causal effects [−0.073 (−0.141, −0.005)]. Estradiol was genetically linked to a lower risk of WHR [−0.637 (−1.092, −0.183)]. SHBG was associated with lower visceral adipose tissue volume [−0.056 (−0.096, −0.016)], WHR [−0.057 (−0.089, −0.025)] and BMI-adjusted WHR [−0.057 (−0.103, −0.011)].

Analysis of obesity-related indicators stratified by sex showed that Bio-T was significantly and negatively associated with BMI in males [−0.068 (−0.134, −0.002)], SHBG was significantly and negatively associated with BMI-adjusted waist circumference [−0.065 (−0.104, −0.025)] and BMI-adjusted WHR [−0.066 (−0.106, −0.027)].

### MR of sex hormones and obesity-related indicators in females

**Fig. 4:**
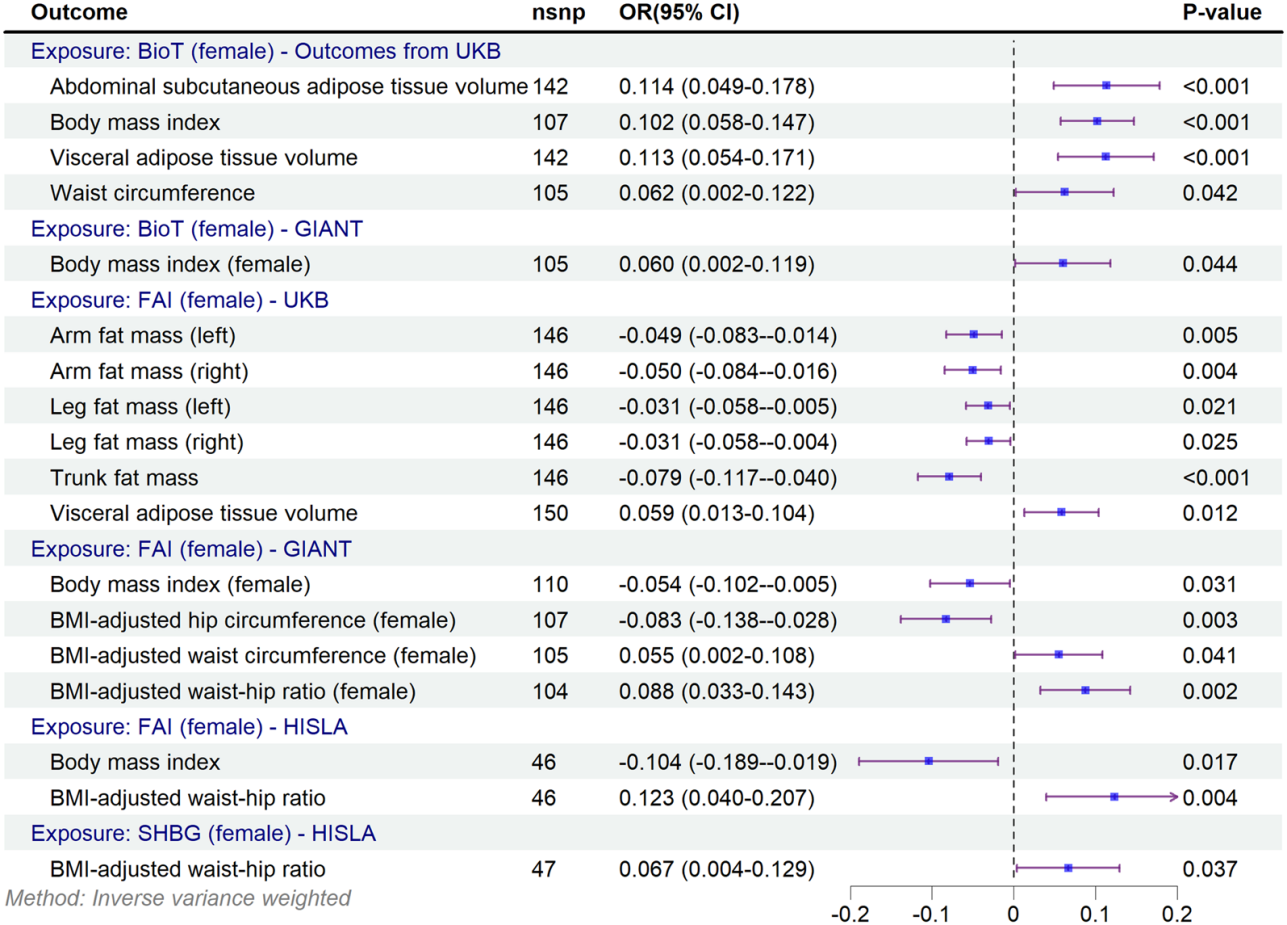
Forest plot of the Mendelian randomization study investigating the effect of sex hormones on obesity-related indicators in females. *BioT* bioavailable testosterone, *BMI* body mass index, *FAI* free androgen index, *SHBG* sex hormone-binding globulin. *P*-values less than 0.05 (*p* < 0.05) were considered significant

Figure 4 illustrates associations between sex hormones and obesity indicators in females, consistent with potential causal relationships. Similar to males, results showed that genetic susceptibility to FAI was significantly associated with lower total body adiposity, but higher visceral adipose tissue volume [0.059 (0.013, 0.104)] and BMI-adjusted WHR [0.123 (0.040, 0.207)] showed significant associations consistent with potential causal effects (Fig. 4). In contrast to males, Bio-T was significantly associated with abdominal subcutaneous adipose tissue volume [0.114 (0.049, 0.178)], visceral adipose tissue volume [0.113 (0. 054, 0.171)], BMI [0.102 (0.058, 0.147)] and waist circumference [0.062 (0.002, 0.122)], which showed a significant positive association consistent with a potential causal effect. SHBG was significantly and positively associated with BMI-adjusted WHR [0.067 (0.004, 0.129)].

In sex-stratified analyses, FAI was significantly positively associated with BMI-adjusted WHR [0.088 (0.033, 0.143)] and BMI-adjusted waist circumference [0.055 (0.002, 0.108)] and significantly positively associated with BMI [−0.054 (−0.102, −0.005)] while BMI-adjusted hip circumference was significantly negatively associated [−0.083 (−0.138, −0.028)]. Similarly, Bio-T was significantly positively associated with BMI [0.060 (0.002, 0.119)].

### Reverse MR of sex hormones and obesity-related indicators in females and males

#### Reverse MR results in females

Reverse MR analysis revealed sex-specific causal associations between obesity-related indicators and sex hormones in females. The key outcomes from the inverse-variance weighting (IVW) analysis are as follows (Figure S12):

BMI exerted a significantly positive causal effect on female BioT [0.245 (0.225, 0.265)]. In contrast, BMI exhibited a causal negative association with female FAI [−0.054 (−0.083, −0.026)], findings consistent with a potential causal effect.

Left leg fat mass had a strong positive effect on female BioT [0.271 (0.234, 0.308)]. Trunk fat mass showed a positive effect on BioT [0.167 (0.136, 0.198)] and a negative effect on FAI [−0.111 (−0.155, −0.068)] simultaneously.

Waist circumference exerted a positive effect on BioT [0.165 (0.091, 0.239)] and a positive effect on sex hormone-binding globulin (SHBG) [4.415 (2.028, 6.802)]. Meanwhile, it showed negative effects on estradiol [−0.254 (−0.426, −0.082)] and FAI [−0.135 (−0.247, −0.022)]. Waist-to-hip ratio (WHR) had a negative effect on estradiol [−0.102 (−0.190, −0.013)] and positive effects on FAI [0.263 (0.189, 0.337)] and SHBG [1.615 (0.152, 3.078)].

#### Reverse MR results in males

Reverse MR analysis in males showed an opposite pattern to that in females: most obesity-related indicators exerted negative effects on androgens (BioT, FAI) and positive effects on estradiol. The specific IVW results are as follows (Figure S13).

Left arm fat mass had a significantly negative association with male BioT [−0.175 (−0.206, −0.144)] and a positive association with estradiol [0.018 (0.011, 0.024)]—both consistent with potential causal effects. Right arm fat mass, left leg fat mass, and right leg fat mass all exhibited consistent trends: negative effects on BioT and FAI, and positive effects on estradiol. Specifically, left leg fat mass had a negative effect on FAI [−0.106 (−0.161, −0.051)] and a positive effect on estradiol [0.021 (0.013, 0.029)].

IVW analysis showed that BMI had a strong negative effect on male BioT [−0.135 (−0.156, −0.114)], along with a positive effect on estradiol [0.014 (0.009, 0.018)] and a negative effect on FAI [−0.051 (−0.082, −0.021)].

Trunk fat mass exerted a negative effect on male BioT [−0.179 (−0.211, −0.147)], a negative effect on FAI [−0.119 (−0.160, −0.077)], and a positive effect on estradiol [0.015 (0.008, 0.021)]. Waist circumference had a negative effect on BioT [−0.121 (−0.214, −0.027)] and a positive effect on estradiol [0.014 (0.003, 0.025)]. WHR showed a negative effect on estradiol [−0.016 (−0.026, −0.006)] and a positive effect on FAI [0.166 (0.079, 0.253)].

### Sensitivity analysis

For detecting potential bias in horizontal pleiotropy, MR-Egger and weighted median were utilized as sensitivity assessments to explore the linkage between sex hormones and –obesity-associated indicators (Figures S10–S11). The results of these methods were analogous to the estimates generated by the IVW approach.

## Discussion

To our knowledge, this research constitutes the first comprehensive examination of the association between sex hormones and obesity-related parameters, combining data from a large observational study and MR analysis of large-scale genetic datasets. In males, higher testosterone, SHBG, and T/E2 ratios were associated with a decreased obesity risk, while estradiol and obesity showed an inverted U-shaped relationship. In females, estradiol and SHBG mitigated obesity-related markers, whereas testosterone and FAI displayed inconsistent or inverted U-shaped trends. MR analyses validated that elevated SHBG and testosterone potentially decreased the risk of obesity in males, while hyperandrogenism increased central obesity risk in females. Multiple sensitivity analyses, employing diverse methodologies, confirmed the robustness of the findings, with consistent results across different analytical strategies.

Total testosterone denotes the entire quantity of testosterone detected in plasma. Roughly 60%–70% of testosterone associates with binding proteins (mainly sex hormone-binding globulin and albumin), forming bound testosterone. These bound testosterone function as stabilizers and reservoirs within the circulatory system. Bio-T represents the portion of testosterone capable of binding to albumin, thereby entering cells via the cell membrane and exerting physiological effects. In males, Bio-T exhibits a negative correlation with nearly all obesity-related markers. Earlier research has indicated that testosterone promotes the decrease of body fat in males [35], a finding that matches our results. Multiple studies have suggested that the impact of testosterone on obesity is multifaceted. Investigations into adipose precursor cells in male rats revealed that testosterone augments catecholamine-induced lipolysis [36]. Additionally, it has been established that androgens suppress adipogenesis in human adipose progenitor cells [37]. Our research also identified a significant association between higher SHBG levels in males and lower visceral fat mass as well as reduced waist circumference. A study on transgenic male mice with human SHBG showed that SHBG can directly stimulate lipolysis in white adipose tissue [38]. Studies have shown that SHBG can bind to LRP1 on adipocyte membranes, activate the cAMP–PKA pathway, and promote triglyceride breakdown [39, 40]. Besides its role in regulating sex hormone levels, SHBG indirectly influences fat metabolism. Cross-sectional analysis further uncovered an inverse U-shaped relationship between estradiol and obesity markers in men. Prior studies have documented a positive correlation between estradiol and BMI, although this association diminishes at higher BMI percentiles [41]. While the exact mechanisms remain unclear, they might be related to the negative feedback inhibition exerted by high estrogen levels on the hypothalamic–pituitary–testicular axis [42]. Aligned with a Nigerian investigation, our analysis identified an inverse relationship between the T/E2 ratio and obesity-associated markers [43]. A key limitation is the non-execution of a reverse MR study. Given that Lee’s research indicated that testosterone undergoes enzymatic conversion to estradiol in adipose tissue, and that increased fat mass may exert a reversing effect on sex hormone levels, future research should prioritize exploring bidirectional biological impacts [44, 45].

In comparison to males, females display lower testosterone concentrations in their blood; however, free androgens hold crucial significance for females, especially those with polycystic ovary syndrome (PCOS). Our findings illustrate that in females, FAI is strongly linked to reduced limb fat mass, higher visceral adipose tissue volume, and an elevated WHR. This also suggests that elevated levels of free androgens tend to induce central obesity in females. Epidemiological investigations have shown that females with PCOS have more severe hyperandrogenism and central obesity [13]. Testosterone can facilitate fat accumulation and insulin resistance in females with PCOS [46]. Moreover, our study discovered that estradiol in females could boost adiposity in the limbs and trunk while decreasing the waist-to-hip ratio. Although this result was not significant in the MR analysis, a significant correlation was found in the cross-sectional analysis. A previous observational study also reported a negative association between estradiol and WHR [47]. Similar to the male results, SHBG had a significant negative correlation with the fat content in the limbs and trunk of females. SHBG is closely associated with lipid metabolism. A recent MR study has indicated that genetically predicted higher SHBG levels may improve lipid profiles by increasing HDL cholesterol levels and decreasing triglyceride levels, which in turn safeguards against coronary atherosclerotic disease [48]. Adiposity is tightly coupled to immunometabolic remodeling. In particular, macrophage glutamine metabolism modulates inflammatory tone within adipose tissue and contributes to insulin resistance and obesity phenotypes. If androgen or SHBG signaling reshapes adipose macrophage programs, this could partially explain the observed associations between sex steroids and central fat depots [49].

The study boasts several notable strengths. Firstly, we integrated NHANES observational datasets with MR methods to probe into the associations between steroid hormones and obesity phenotypes. Secondly, NHANES is a large-scale, population-based study in the US. We made use of 16 obesity-related metrics as outcomes. Besides the traditional waist circumference and BMI, we zeroed in on indicators like android/gynoid obesity and also looked into the linear and non-linear relationships between steroid hormones and obesity-related indicators. Thirdly, in MR analyses, using genetic variation as an instrumental variable makes the study less prone to reverse causality and confounders, and the sample size is fairly objective. There are also some limitations worth noting. Firstly, due to our inability to obtain repeated hormone level measurements from the participants, we relied solely on questionnaires and self-reports to determine menopausal status, which introduces a certain degree of recall bias. Secondly, there is a certain amount of heterogeneity in the outcome data. Fortunately, the random-effects IVW model we selected can overlook the precision loss caused by this heterogeneity. Second, owing to variations in allele frequencies and disease/exposure prevalence across distinct ancestral groups, IVs originating from European populations might be absent in other cohorts. This circumstance confines the scope of the genetic analysis to individuals of European descent. Going forward, these results should be validated through a prospective cohort study conducted within multi-ethnic groups to address the limitations imposed by ancestral disparities in IV applicability. Thirdly, we clarify GAM’s role in our study as a visualization tool for potential non-linear trends, acknowledging its inability to formalize non-linearity testing, and suggesting that future studies could complement RCS analysis to validate these observed U-shaped associations. Fourth, defining “premenopausal women” solely by self-reported “having menstrual periods in the past 12 months” may misclassify perimenopausal women (aged 45–55 years). Their fluctuating sex hormone levels could dilute true associations between sex hormones and obesity indicators, reducing the precision of female subgroup results. Future studies should integrate biochemical markers (e.g., FSH) with menstrual history for accurate classification.

## Conclusions

To summarize, the present study uncovered sex-specific connections between sex hormones and obesity-related indicators. Among males, androgens and SHBG showed a significant negative correlation with overall adiposity, while estradiol had a non-linear correlation with obesity. In females, hyperandrogenism was closely linked to obesity, particularly central obesity, often leading to an apple-shaped physique. Based on these results, we propose sex-specific interventions: males prioritize SHBG modulation to reduce visceral fat, and testosterone replacement for low Bio-T. Females (especially PCOS) use androgen-lowering therapies to cut central obesity risk, with DXA limb fat monitoring as an early warning. Further research is required to delve deeper into the underlying biological mechanisms.

## Supplementary Information

Supplementary material 1.

## Data Availability

The datasets of NHANES in this study are available at https://wwwn.cdc.gov/nchs/nhanes. The datasets of MR can be found in Table S1.

## Abbreviations

A/G: Android and gynoid
Bio-T: Bioavailable testosterone
BMI: Body mass index
CDC: National Center for Disease Control and Prevention
CVD: Cardiovascular disease’s risk factors
DXA: Dual-energy X-ray absorptiometry
FAI: Free androgen index
GIANGT: Genetic investigation of anthropometric traits
HEI: Healthy eating index
HISLA: Hispanic/Latino anthropometry
IV: Instrumental variable
IVW: Inverse variance weighting
LD: Linkage disequilibrium
LTPA: Leisure time physical activity
MEC: Mobile examination center
MR: Mendelian randomization
NCHS: National Center for Health Statistics
NHANES: National Health and Nutrition Examination Survey
PCOS: Polycystic ovary syndrome
SAT: Subcutaneous adipose tissue
SHBG: Sex hormone-binding globulin
SNP: Single nucleotide polymorphism
T2D: Type 2 diabetes
T/E2: Testosterone to estradiol
UKB: UK Biobank
U.S.: United States
VAT: Visceral adipose tissue
WHO: World Health Organization
WHR: Waist-to-hip ratio

## References

[1] Vecchié A, Dallegri F, Carbone F, Bonaventura A, Liberale L, Portincasa P, et al. Obesity phenotypes and their paradoxical association with cardiovascular diseases. Eur J Intern Med. 2018;48:6–17. 10.1016/j.ejim.2017.10.020. 29100895 10.1016/j.ejim.2017.10.020

[2] Hales CM, Carroll MD, Fryar CD, Ogden CL. Prevalence of obesity and severe obesity among adults: United States, 2017–2018. NCHS Data Brief. 2020. 1–8. 32487284

[3] Tahrani AA, Morton J. Benefits of weight loss of 10% or more in patients with overweight or obesity: a review. Obesity. 2022;30:802–40. 10.1002/oby.23371. 35333446 10.1002/oby.23371

[4] F M, S M, Br K, F R-J, S S. Adipokines at the crossroad between obesity and cardiovascular disease. https://pubmed.ncbi.nlm.nih.gov/25338625/. Accessed 25 Sept 2025

[5] Loh NY, Humphreys E, Karpe F, Tomlinson JW, Noordam R, Christodoulides C. Sex hormones, adiposity, and metabolic traits in men and women: a Mendelian randomisation study. Eur J Endocrinol. 2022;186:407–16. 10.1530/EJE-21-0703. 35049520 10.1530/EJE-21-0703 PMC8859921

[6] Ee K, Js F. Adipose tissue as an endocrine organ. https://pubmed.ncbi.nlm.nih.gov/15181022/. Accessed 25 Sept 2025

[7] F B, U E, M A, S H, L P, Yh C, et al. Adipose tissue dysfunction in polycystic ovary syndrome. 2023. https://pubmed.ncbi.nlm.nih.gov/37329216/. Accessed 25 Sept 2025

[8] S M-I, M H, A T-K, M T, K K, M K, et al. Adipose tissue insulin resistance index was inversely associated with gluteofemoral fat and skeletal muscle mass in Japanese women. 2024. https://pubmed.ncbi.nlm.nih.gov/39013950/. Accessed 25 Sept 2025 10.1038/s41598-024-67184-6 PMC11252386 39013950

[9] Fan S, Chen S, Lin L. Research progress of gut microbiota and obesity caused by high-fat diet. Front Cell Infect Microbiol. 2023;13:1139800. 10.3389/fcimb.2023.1139800. 36992691 10.3389/fcimb.2023.1139800 PMC10040832

[10] Xu F, Earp JE, LoBuono DL, Greene GW. The relationship of physical activity and dietary quality with android fat composition and distribution in US adults. Nutrients. 2022;14:2804. 10.3390/nu14142804. 35889761 10.3390/nu14142804 PMC9318818

[11] Kelly DM, Jones TH. Testosterone and obesity. Obes Rev. 2015;16:581–606. 10.1111/obr.12282. 25982085 10.1111/obr.12282

[12] Gibson DA, Saunders PTK. Endocrine disruption of oestrogen action and female reproductive tract cancers. Endocr Relat Cancer. 2014;21:T13-31. 10.1530/ERC-13-0342. 24163391 10.1530/ERC-13-0342

[13] Zhang H, Wang W, Zhao J, Jiao P, Zeng L, Zhang H, et al. Relationship between body composition, insulin resistance, and hormonal profiles in women with polycystic ovary syndrome. Front Endocrinol. 2022;13:1085656. 10.3389/fendo.2022.1085656. 10.3389/fendo.2022.1085656 PMC9869160 36699018

[14] Kim D, Manikat R, Cholankeril G, Ahmed A. Endogenous sex hormones and nonalcoholic fatty liver disease in US adults. Liver Int. 2024;44:460–71. 10.1111/liv.15786. 38010926 10.1111/liv.15786

[15] Huebschmann AG, Huxley RR, Kohrt WM, Zeitler P, Regensteiner JG, Reusch JEB. Sex differences in the burden of type 2 diabetes and cardiovascular risk across the life course. Diabetologia. 2019;62:1761–72. 10.1007/s00125-019-4939-5. 31451872 10.1007/s00125-019-4939-5 PMC7008947

[16] Braunstein GD. Clinical practice. Gynecomastia. N Engl J Med. 2007;357:1229–37. 10.1056/NEJMcp070677. 17881754 10.1056/NEJMcp070677

[17] Grossmann M, Wierman ME, Angus P, Handelsman DJ. Reproductive endocrinology of nonalcoholic fatty liver disease. Endocr Rev. 2019;40:417–46. 10.1210/er.2018-00158. 30500887 10.1210/er.2018-00158

[18] van Koeverden ID, de Bakker M, Haitjema S, van der Laan SW, de Vries J-PPM, Hoefer IE, et al. Testosterone to oestradiol ratio reflects systemic and plaque inflammation and predicts future cardiovascular events in men with severe atherosclerosis. Cardiovasc Res. 2019;115:453–62. 10.1093/cvr/cvy188. 30052805 10.1093/cvr/cvy188

[19] Keevil BG, Adaway J. Assessment of free testosterone concentration. J Steroid Biochem Mol Biol. 2019;190:207–11. 10.1016/j.jsbmb.2019.04.008. 30970279 10.1016/j.jsbmb.2019.04.008

[20] Genotypic and phenotypic spectrum of CCDC141 variants in a Chinese cohort with congenital hypogonadotropic hypogonadism | European Journal of Endocrinology | Oxford Academic. https://academic.oup.com/ejendo/article-abstract/183/3/245/6653722?redirectedFrom=fulltext. Accessed 30 Oct 2025 10.1530/EJE-19-1018 32520725

[21] Gagnon E, Mitchell PL, Arsenault BJ. Body fat distribution, fasting insulin levels, and insulin secretion: a bidirectional Mendelian randomization study. J Clin Endocrinol Metab. 2023;108:1308–17. 10.1210/clinem/dgac758. 36585897 10.1210/clinem/dgac758

[22] Wan B, Ma N, Zhou Z, Lv C. Putative causal inference for the relationship between obesity and sex hormones in males: a bidirectional Mendelian randomization study. PeerJ. 2023;11:e15760. 10.7717/peerj.15760. 37483981 10.7717/peerj.15760 PMC10362853

[23] Gao Y, Wang C, Wang K, He C, Hu K, Liang M. The effects and molecular mechanism of heat stress on spermatogenesis and the mitigation measures. Syst Biol Reprod Med. 2022;68:331–47. 10.1080/19396368.2022.2074325. 35722894 10.1080/19396368.2022.2074325

[24] Li H, Xu X, Wang X, Liao X, Li L, Yang G, et al. Free androgen index and Irisin in polycystic ovary syndrome. J Endocrinol Invest. 2016;39:549–56. 10.1007/s40618-015-0403-7. 26584566 10.1007/s40618-015-0403-7

[25] Zhang Y-B, Chen C, Pan X-F, Guo J, Li Y, Franco OH, et al. Associations of healthy lifestyle and socioeconomic status with mortality and incident cardiovascular disease: two prospective cohort studies. BMJ. 2021. 10.1136/bmj.n604. 33853828 10.1136/bmj.n604 PMC8044922

[26] Cao C, Friedenreich CM, Yang L. Association of daily sitting time and leisure-time physical activity with survival among US cancer survivors. JAMA Oncol. 2022;8:395–403. 10.1001/jamaoncol.2021.6590. 34989765 10.1001/jamaoncol.2021.6590 PMC8739832

[27] Leinonen JT, Mars N, Lehtonen LE, Ahola-Olli A, Ruotsalainen S, Lehtimäki T, et al. Genetic analyses implicate complex links between adult testosterone levels and health and disease. Commun Med. 2023;3:4. 10.1038/s43856-022-00226-0. 36653534 10.1038/s43856-022-00226-0 PMC9849476

[28] Ruth KS, Day FR, Tyrrell J, Thompson DJ, Wood AR, Mahajan A, et al. Using human genetics to understand the disease impacts of testosterone in men and women. Nat Med. 2020;26:252–8. 10.1038/s41591-020-0751-5. 32042192 10.1038/s41591-020-0751-5 PMC7025895

[29] Schmitz D, Ek WE, Berggren E, Höglund J, Karlsson T, Johansson Å. Genome-wide association study of estradiol levels and the causal effect of estradiol on bone mineral density. J Clin Endocrinol Metab. 2021;106:e4471–86. 10.1210/clinem/dgab507. 34255042 10.1210/clinem/dgab507 PMC8530739

[30] Koskeridis F, Evangelou E, Said S, Boyle JJ, Elliott P, Dehghan A, et al. Pleiotropic genetic architecture and novel loci for C-reactive protein levels. Nat Commun. 2022;13:6939. 10.1038/s41467-022-34688-6. 36376304 10.1038/s41467-022-34688-6 PMC9663411

[31] Shungin D, Winkler TW, Croteau-Chonka DC, Ferreira T, Locke AE, Mägi R, et al. New genetic loci link adipose and insulin biology to body fat distribution. Nature. 2015;518:187–96. 10.1038/nature14132. 25673412 10.1038/nature14132 PMC4338562

[32] Loh P-R, Kichaev G, Gazal S, Schoech AP, Price AL. Mixed-model association for biobank-scale datasets. Nat Genet. 2018;50:906–8. 10.1038/s41588-018-0144-6. 29892013 10.1038/s41588-018-0144-6 PMC6309610

[33] Fernández-Rhodes L, Graff M, Buchanan VL, Justice AE, Highland HM, Guo X, et al. Ancestral diversity improves discovery and fine-mapping of genetic loci for anthropometric traits-The Hispanic/Latino Anthropometry Consortium. Hum Genet Genomics Adv. 2022;3:100099. 10.1016/j.xhgg.2022.100099. 10.1016/j.xhgg.2022.100099 PMC8990175 35399580

[34] Li L, Sun Y, Luo J, Liu M. Frontiers | Circulating immune cells and risk of osteosarcoma: a Mendelian randomization analysis. Front Immunol. 10.3389/fimmu.2024.1381212 10.3389/fimmu.2024.1381212 PMC11286390 39081321

[35] Mohammadi-Shemirani P, Chong M, Pigeyre M, Morton RW, Gerstein HC, Paré G. Effects of lifelong testosterone exposure on health and disease using Mendelian randomization. Elife. 2020;9:e58914. 10.7554/eLife.58914. 33063668 10.7554/eLife.58914 PMC7591257

[36] Xu X, De Pergola G, Björntorp P. The effects of androgens on the regulation of lipolysis in adipose precursor cells. Endocrinology. 1990;126:1229–34. 10.1210/endo-126-2-1229. 2153523 10.1210/endo-126-2-1229

[37] Tchernof A, Brochu D, Maltais-Payette I, Mansour MF, Marchand GB, Carreau A-M, et al. Androgens and the regulation of adiposity and body fat distribution in humans. Compr Physiol. 2018. 10.1002/cphy.c170009. 30215860 10.1002/cphy.c170009

[38] Saez-Lopez C, Villena JA, Simó R, Selva DM. Sex hormone-binding globulin overexpression protects against high-fat diet-induced obesity in transgenic male mice. J Nutr Biochem. 2020;85:108480. 10.1016/j.jnutbio.2020.108480. 32795655 10.1016/j.jnutbio.2020.108480

[39] H Y, A K, H S, M F, T Y, Y N, et al. Protective effect of sex hormone-binding globulin against metabolic syndrome: in vitro evidence showing anti-inflammatory and lipolytic effects on adipocytes and macrophages. 2018. https://pubmed.ncbi.nlm.nih.gov/30046278/. Accessed 25 Sept 2025 10.1155/2018/3062319 PMC6036814 30046278

[40] C S-L, Ja V, R S, Dm S. Sex hormone-binding globulin overexpression protects against high-fat diet-induced obesity in transgenic male mice. https://pubmed.ncbi.nlm.nih.gov/32795655/. Accessed 25 Sept 2025 10.1016/j.jnutbio.2020.108480 32795655

[41] Lv X, Jiang Y-T, Zhang X-Y, Li L-L, Zhang H-G, Liu R-Z. Associations of sex hormone levels with body mass index (BMI) in men: a cross-sectional study using quantile regression analysis. Asian J Androl. 2022;25:98–102. 10.4103/aja202212. 10.4103/aja202212 PMC9933972 35439874

[42] Raven G, de Jong FH, Kaufman J-M, de Ronde W. In men, peripheral estradiol levels directly reflect the action of estrogens at the hypothalamo-pituitary level to inhibit gonadotropin secretion. J Clin Endocrinol Metab. 2006;91:3324–8. 10.1210/jc.2006-0462. 16787981 10.1210/jc.2006-0462

[43] Olasore HSA, Oyedeji TA, Olawale MO, Ogundele OI, Faleti JO-O. Relationship between testosterone–estradiol ratio and some anthropometric and metabolic parameters among Nigerian men. Metab Open. 2023;18:100249. 10.1016/j.metop.2023.100249. 10.1016/j.metop.2023.100249 PMC10313505 37396673

[44] Palmisano BT, Zhu L, Eckel RH, Stafford JM. Sex differences in lipid and lipoprotein metabolism. Mol Metab. 2018;15:45–55. 10.1016/j.molmet.2018.05.008. 29858147 10.1016/j.molmet.2018.05.008 PMC6066747

[45] Lee H-K, Lee JK, Cho B. The role of androgen in the adipose tissue of males. World J Mens Health. 2013;31:136–40. 10.5534/wjmh.2013.31.2.136. 24044108 10.5534/wjmh.2013.31.2.136 PMC3770848

[46] O’Reilly MW, Kempegowda P, Walsh M, Taylor AE, Manolopoulos KN, Allwood JW, et al. AKR1C3-mediated adipose androgen generation drives lipotoxicity in women with polycystic ovary syndrome. J Clin Endocrinol Metab. 2017;102:3327–39. 10.1210/jc.2017-00947. 28645211 10.1210/jc.2017-00947 PMC5587066

[47] Mondragón-Ceballos R, García Granados MD, Cerda-Molina AL, Chavira-Ramírez R, Hernández-López LE. Waist-to-hip ratio, but not body mass index, is associated with testosterone and estradiol concentrations in young women. Int J Endocrinol. 2015;2015:654046. 10.1155/2015/654046. 26351453 10.1155/2015/654046 PMC4553330

[48] Yuan S, Wang L, Sun J, Yu L, Zhou X, Yang J, et al. Genetically predicted sex hormone levels and health outcomes: phenome-wide Mendelian randomization investigation. Int J Epidemiol. 2022;51:1931–42. 10.1093/ije/dyac036. 35218343 10.1093/ije/dyac036 PMC9749729

[49] Glutamine metabolism in macrophages: a novel target for obesity/type 2 diabetes. 10.1093/advances/nmy084 10.1093/advances/nmy084 PMC6416106 30753258

