# Supplementary material for: Associations between sex hormones and obesity-related indicators: results from the NHANES and Mendelian randomization study
Source: Eur J Med Res. 2025 Nov 27;30:1216. doi: 10.1186/s40001-025-03470-x (PMC12683816; doi:10.1186/s40001-025-03470-x)
Supplement: Supplementary file 1 — Supplementary material 1. [file 40001_2025_3470_MOESM1_ESM.pdf]

Contents

Figure S1. Overall study design based on observational analysis and Mendelian randomization. 3

Figure S2. Flowchart of participant selection process in the NHANES 2013-2016 analysis of sex hormones and fat distribution. 4

Figure S3. Mendelian randomization assumptions and criteria for valid instrumental variables linking sex hormones to obesity-related indicators. 5

Figure S4. Smoothed curves derived from a generalized additive model illustrating the relationship between estradiol (continuous variable) and 16 obesity-related indicators (continuous variable). 6

Figure S5. Smoothed curves derived from a generalized additive model illustrating the relationship between sex hormone-binding globulin (continuous variable) and 16 obesity-related indicators (continuous variable). 7

Figure S6. Smoothed curves derived from a generalized additive model illustrating the relationship between testosterone-to-estradiol ratio (continuous variable) and 16 obesity-related indicators (continuous variable). 8

Figure S7. Smoothed curves derived from a generalized additive model illustrating the relationship between estradiol (continuous variable) and 16 obesity-related indicators (continuous variable). 9

Figure S8. Smoothed curves derived from a generalized additive model illustrating the relationship between total testosterone (continuous variable) and 16 obesity-related indicators (continuous variable). 10

Figure S9. Smoothed curves derived from a generalized additive model illustrating the relationship between sex hormone-binding globulin (continuous variable) and 16 obesity-related indicators (continuous variable). 11

Figure S10. Forest plot of the Mendelian randomization study investigating the effect of sex hormones on obesity-related indicators in males. 12

Figure S11. Forest plot of the Mendelian randomization study investigating the effect of sex hormones on obesity-related indicators in females. 13

Figure S12. Forest plot of the Mendelian randomization study investigating the effect of obesity-related indicators on sex hormones in females. 14

Figure S13. Forest plot of the Mendelian randomization study investigating the effect of obesity-related indicators on sex hormones in males. 15

Table S1. Detailed data of GWAS studies included in the MR analyses. 16

Table S2. Association of estradiol with 16 obesity-related indicators in males from NHANES 2013-2016 (n=2870). 19

Table S3. Association of SHBG with 16 obesity-related indicators in males from NHANES 2013-2016 (n=2870). 23

Table S4. Association of estradiol with 16 obesity-related indicators in females from NHANES 2013-2016 (n=2309). 27

Table S5. Association of SHBG with 16 obesity-related indicators in females from NHANES 2013-2016 (n=2309). 31

Table S6. nSNP under different P-value thresholds. 35

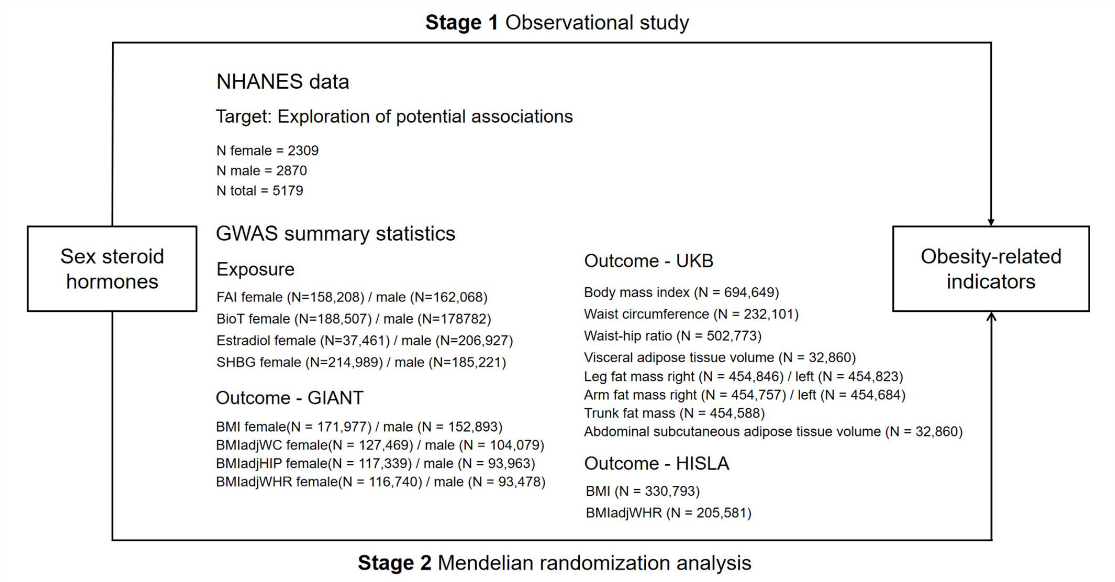

Figure S1. Overall study design based on observational analysis and Mendelian randomization.

Note: Abbreviations: BioT, bioavailable testosterone; BMI, body mass index; BMIadjHIP, BMI-adjusted hip circumference; BMIadjWC, BMI-adjusted waist circumference; BMIadjWHR, BMI-adjusted waist-hip ratio; FAI, free androgen index; N, number of participants; SHBG, single nucleotide polymorphisms

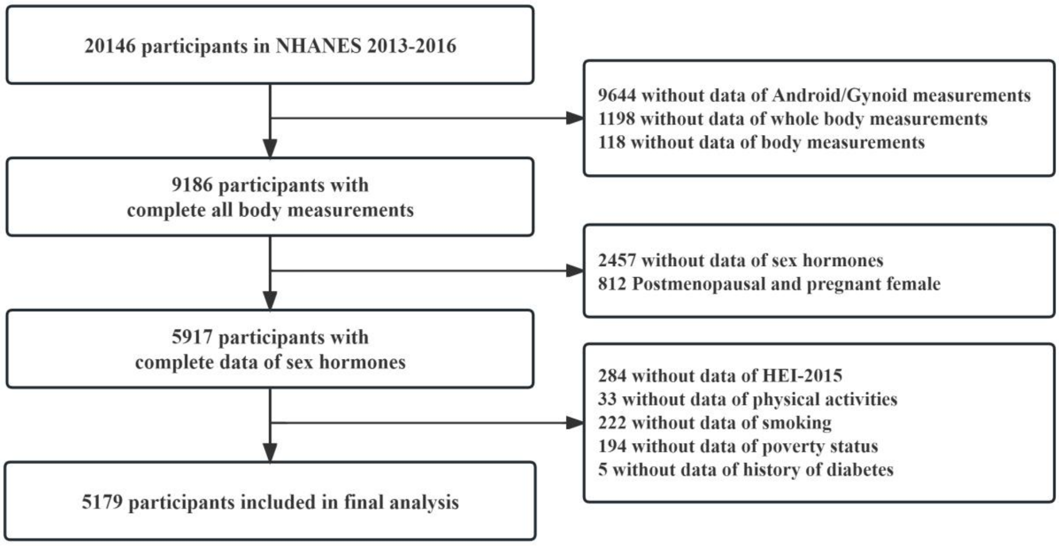

Figure S2. Flowchart of participant selection process in the NHANES 2013-2016 analysis of sex hormones and fat distribution.

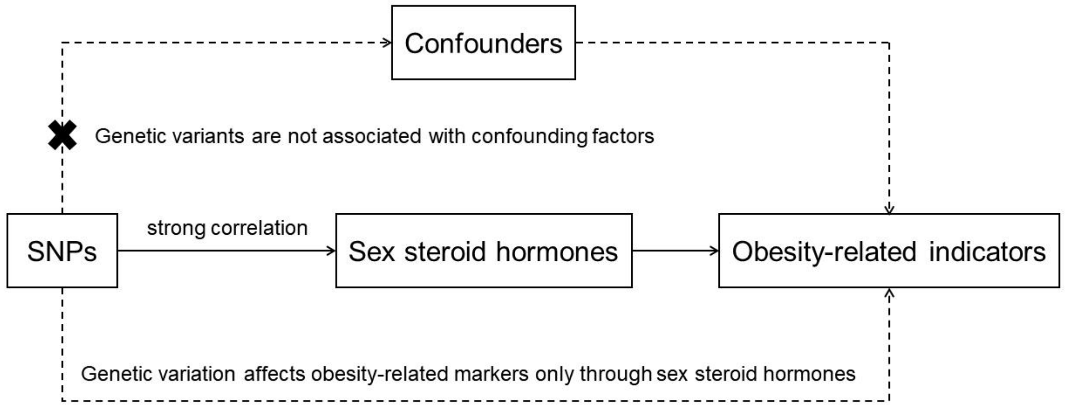

Figure S3. Mendelian randomization assumptions and criteria for valid instrumental variables linking sex hormones to obesity-related indicators.

Note: Abbreviations: SNP, single nucleotide polymorphism.

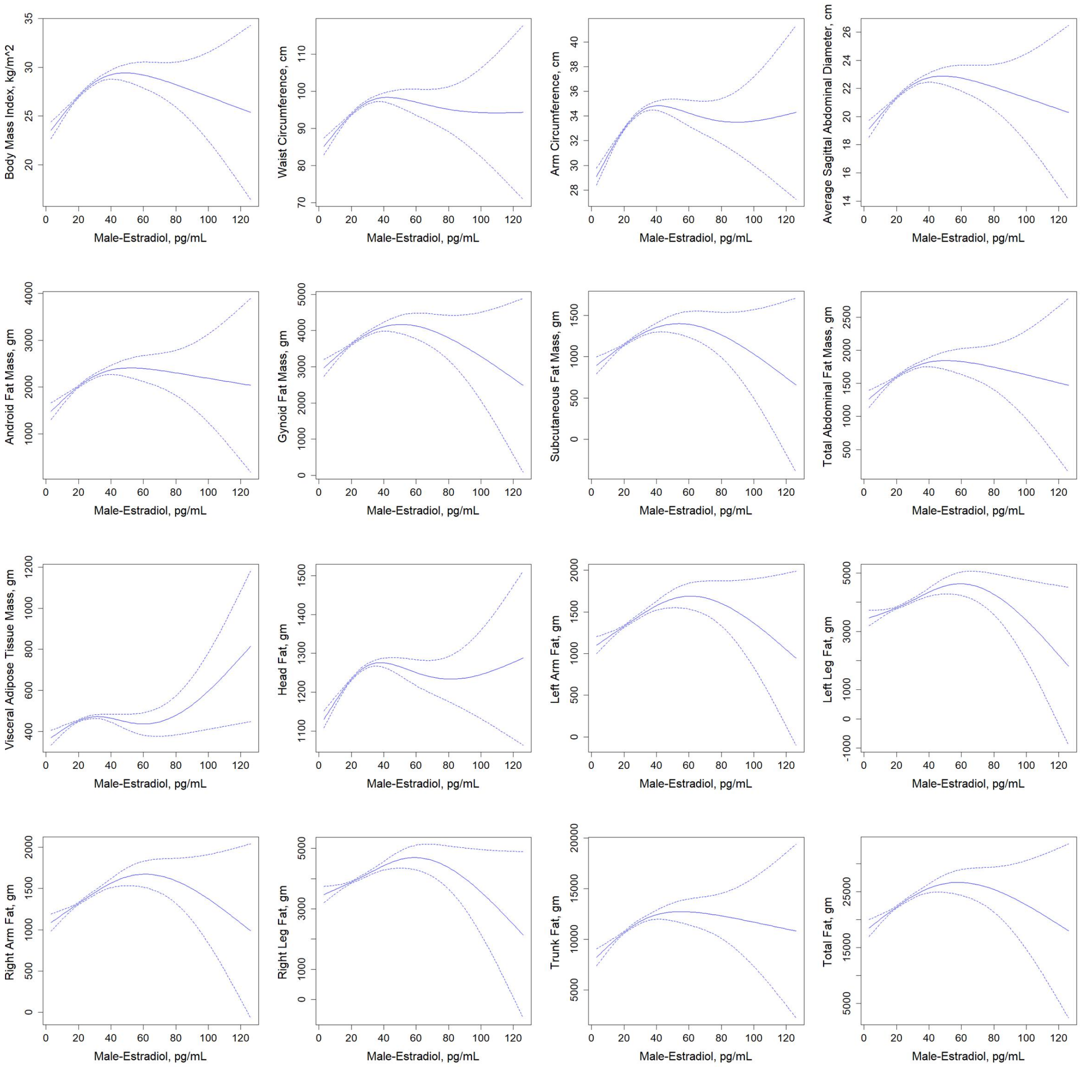

Figure S4. Smoothed curves derived from a generalized additive model illustrating the relationship between estradiol (continuous variable) and 16 obesity-related indicators (continuous variable).

Note: Model was adjusted for age, ethnicity, education level, poverty status, smoking status, HEI-2015, alcohol consumption and leisure-time physical activity.

Abbreviations: gm, gram.

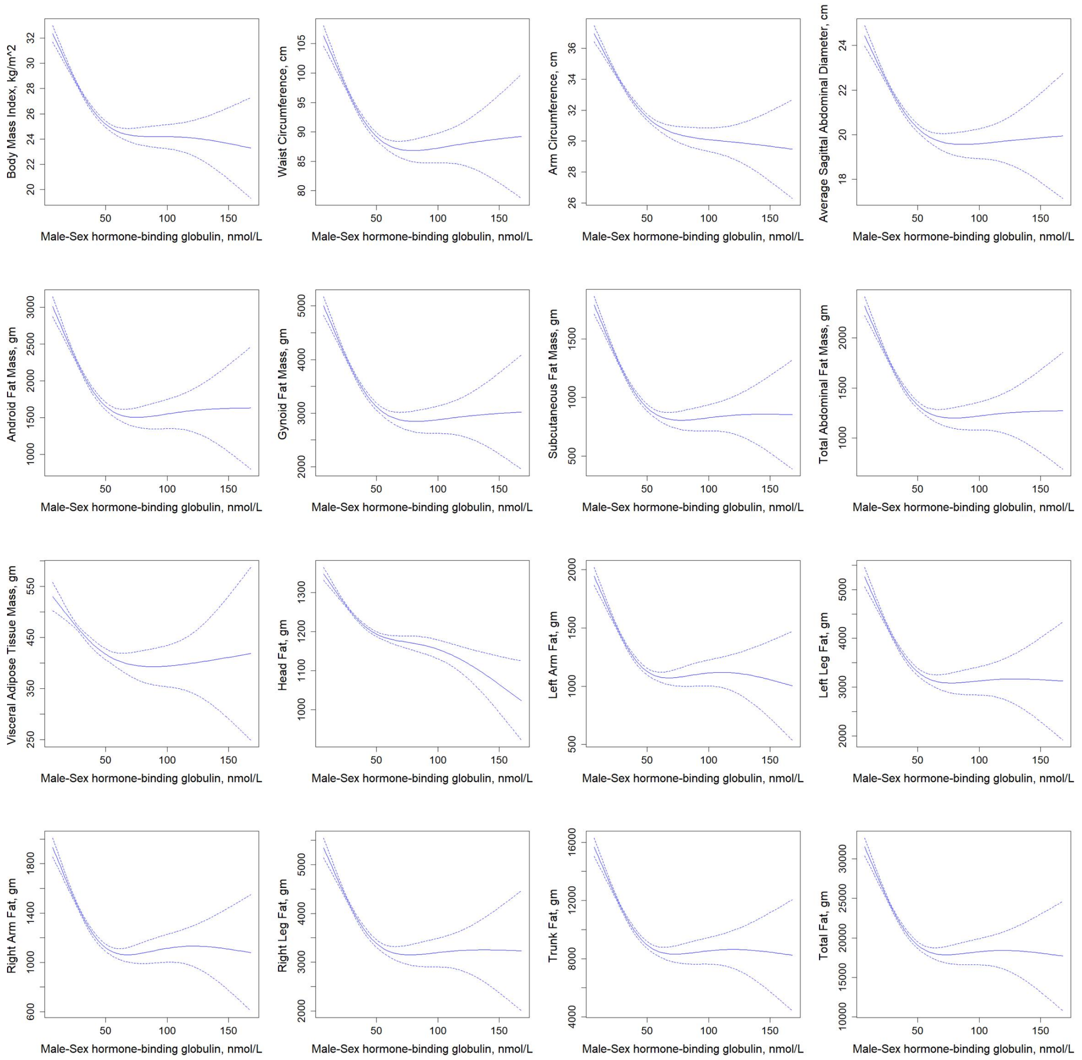

Figure S5. Smoothed curves derived from a generalized additive model illustrating the relationship between sex hormone-binding globulin (continuous variable) and 16 obesity-related indicators (continuous variable).

Note: Model was adjusted for age, ethnicity, education level, poverty status, smoking status, HEI-2015, alcohol consumption and leisure-time physical activity.

Abbreviations: gm, gram.

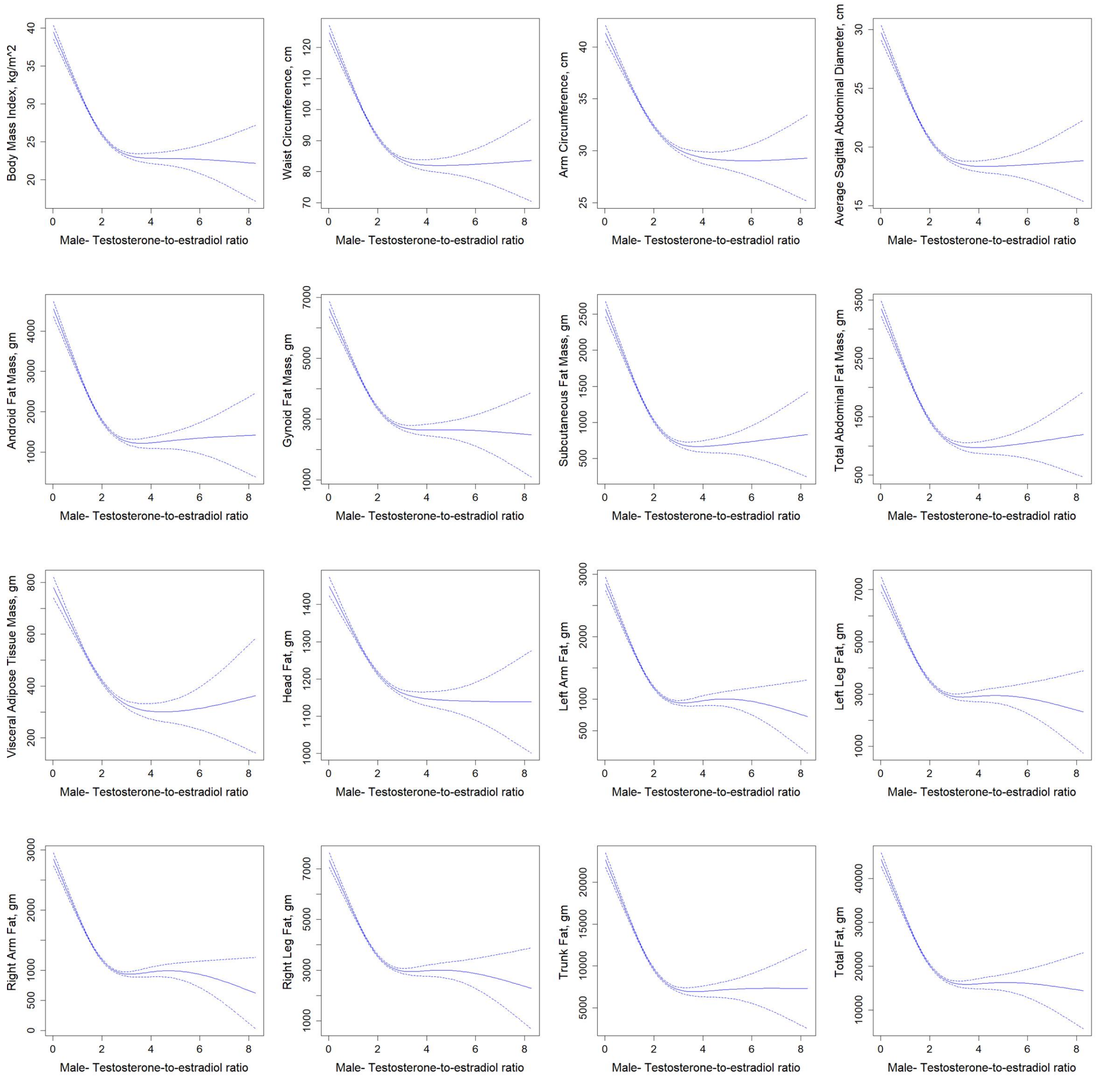

Figure S6. Smoothed curves derived from a generalized additive model illustrating the relationship between testosterone-to-estradiol ratio (continuous variable) and 16 obesity-related indicators (continuous variable).

Note: Model was adjusted for age, ethnicity, education level, poverty status, smoking status, HEI-2015, alcohol consumption and leisure-time physical activity.

Abbreviations: gm, gram.

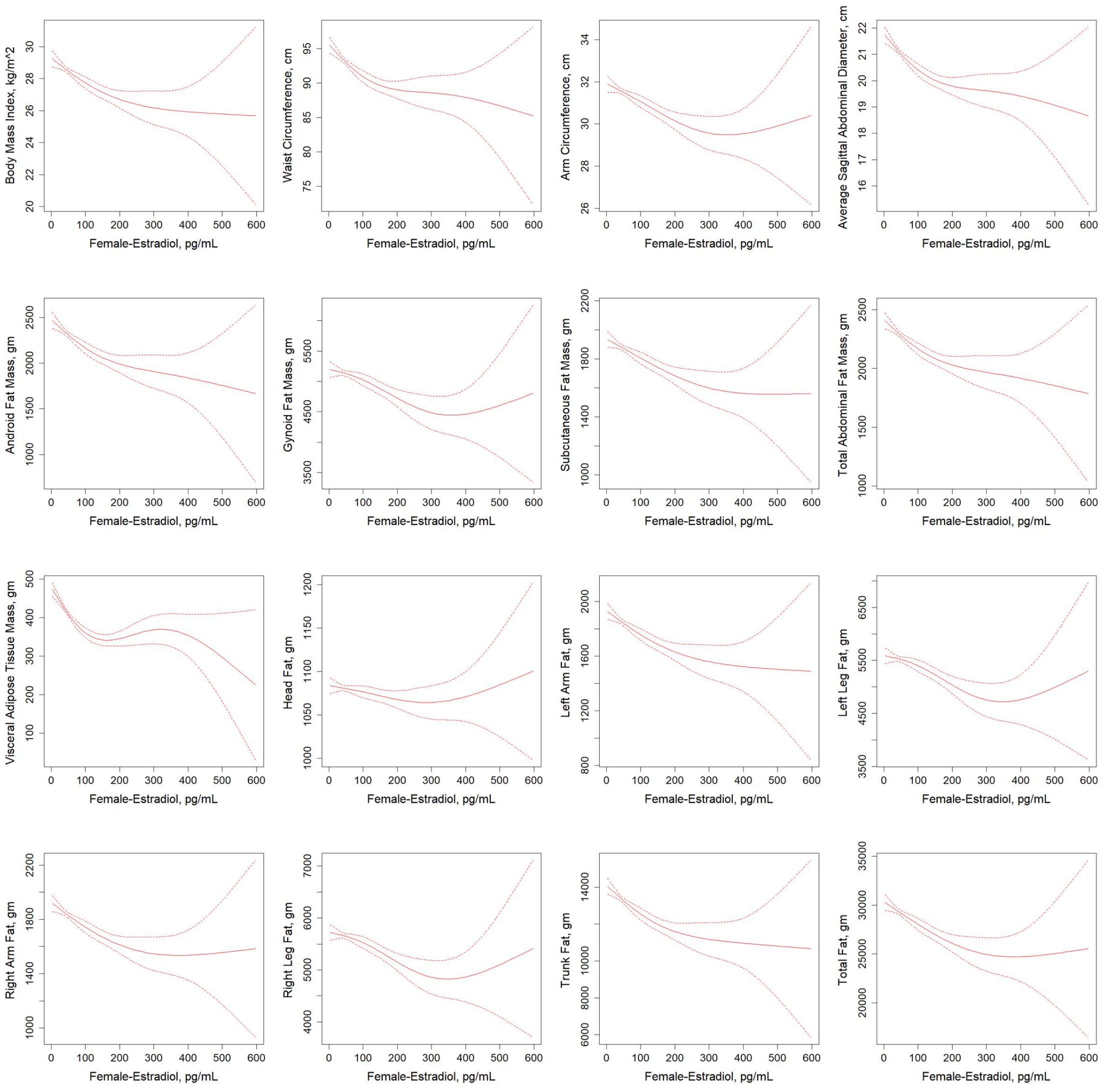

Figure S7. Smoothed curves derived from a generalized additive model illustrating the relationship between estradiol (continuous variable) and 16 obesity-related indicators (continuous variable).

Note: Model was adjusted for age, ethnicity, education level, poverty status, smoking status, HEI-2015, alcohol consumption and leisure-time physical activity.

Abbreviations: gm, gram.

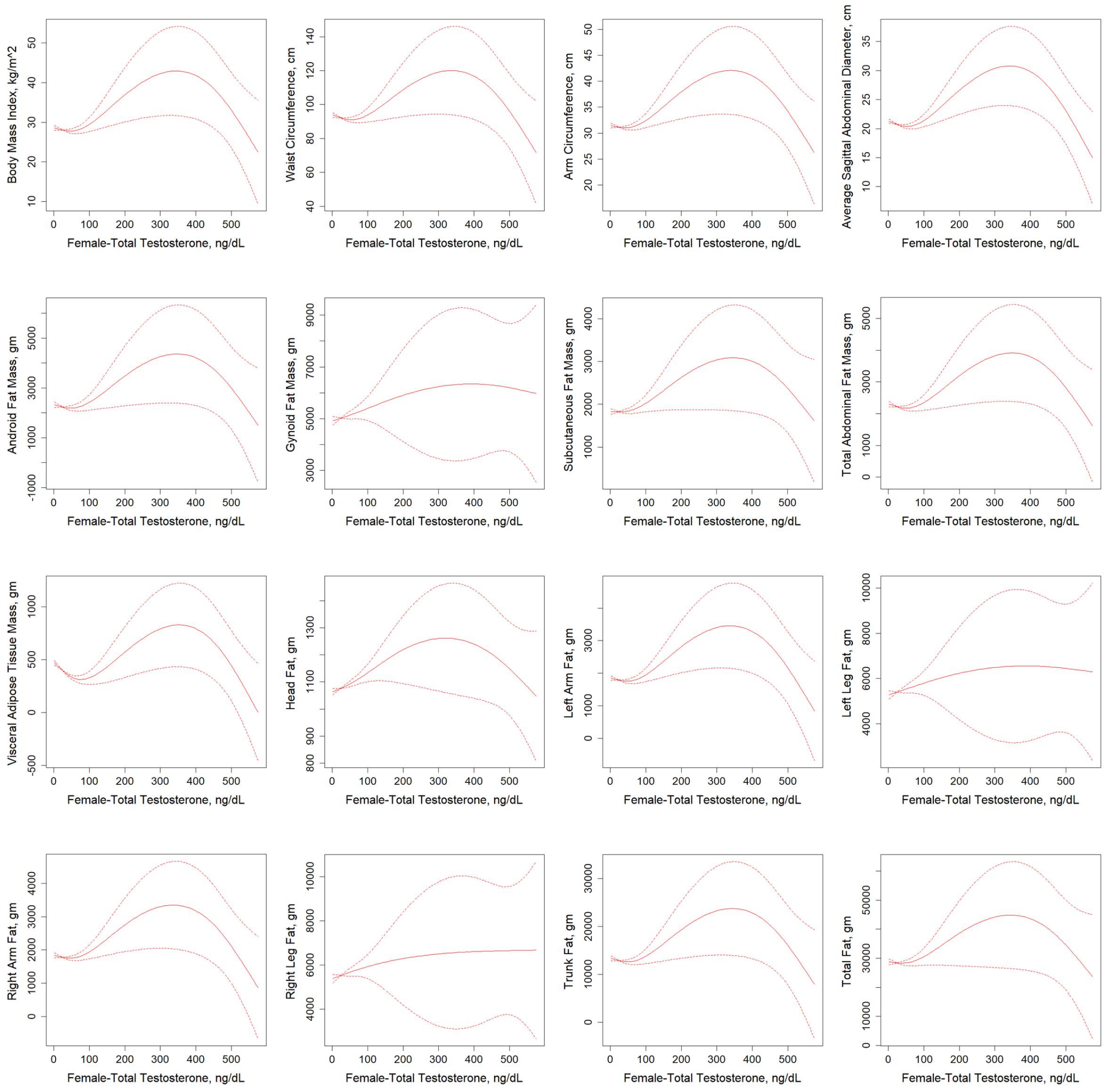

Figure S8. Smoothed curves derived from a generalized additive model illustrating the relationship between total testosterone (continuous variable) and 16 obesity-related indicators (continuous variable).

Note: Model was adjusted for age, ethnicity, education level, poverty status, smoking status, HEI-2015, alcohol consumption and leisure-time physical activity.

Abbreviations: gm, gram.

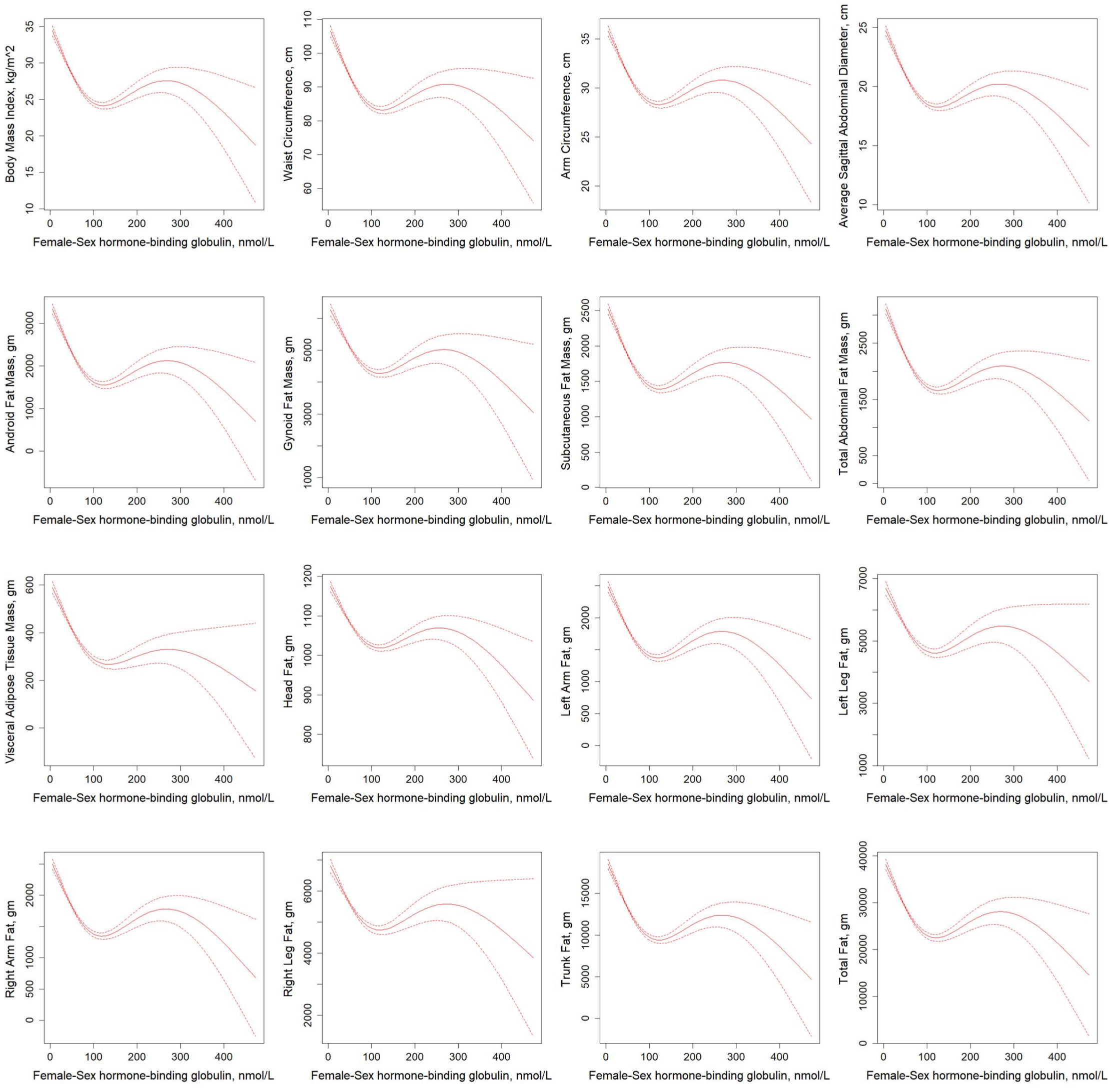

Figure S9. Smoothed curves derived from a generalized additive model illustrating the relationship between sex hormone-binding globulin (continuous variable) and 16 obesity-related indicators (continuous variable).

Note: Model was adjusted for age, ethnicity, education level, poverty status, smoking status, HEI-2015, alcohol consumption and leisure-time physical activity.

Abbreviations: gm, gram.

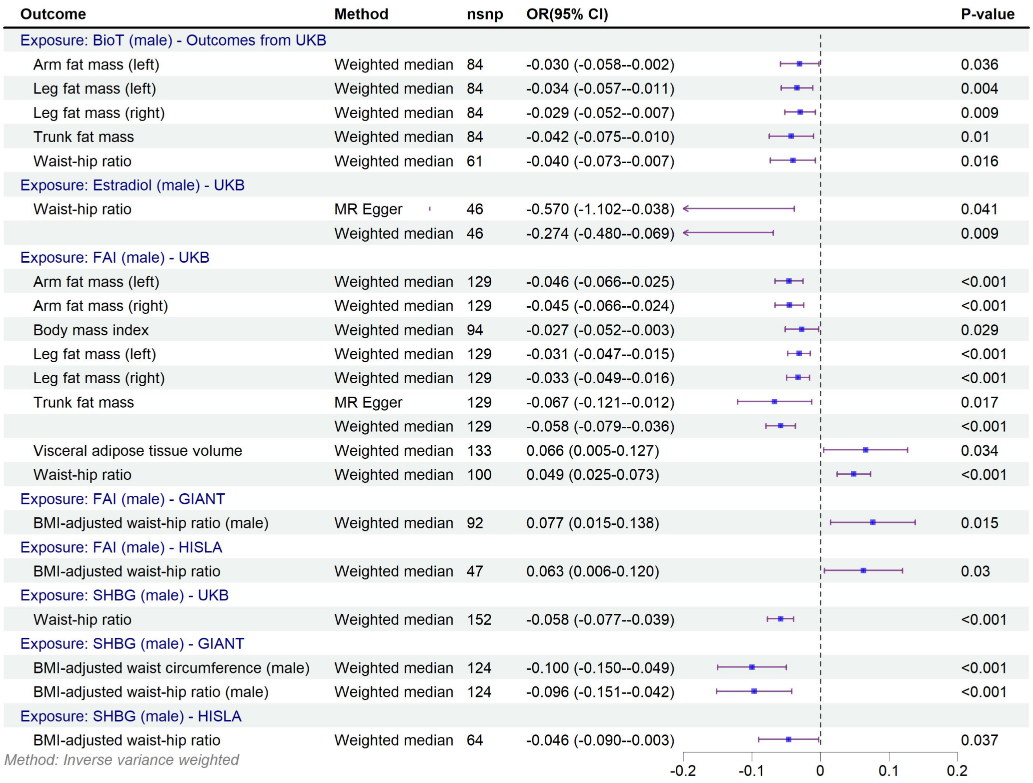

Figure S10. Forest plot of the Mendelian randomization study investigating the effect of sex hormones on obesity-related indicators in males.

Note: Abbreviations: BioT, bioavailable testosterone; BMI, body mass index; FAI, free androgen index; SHBG, single nucleotide polymorphisms. P-values less than 0.05 ($p < 0.05$) were considered significant.

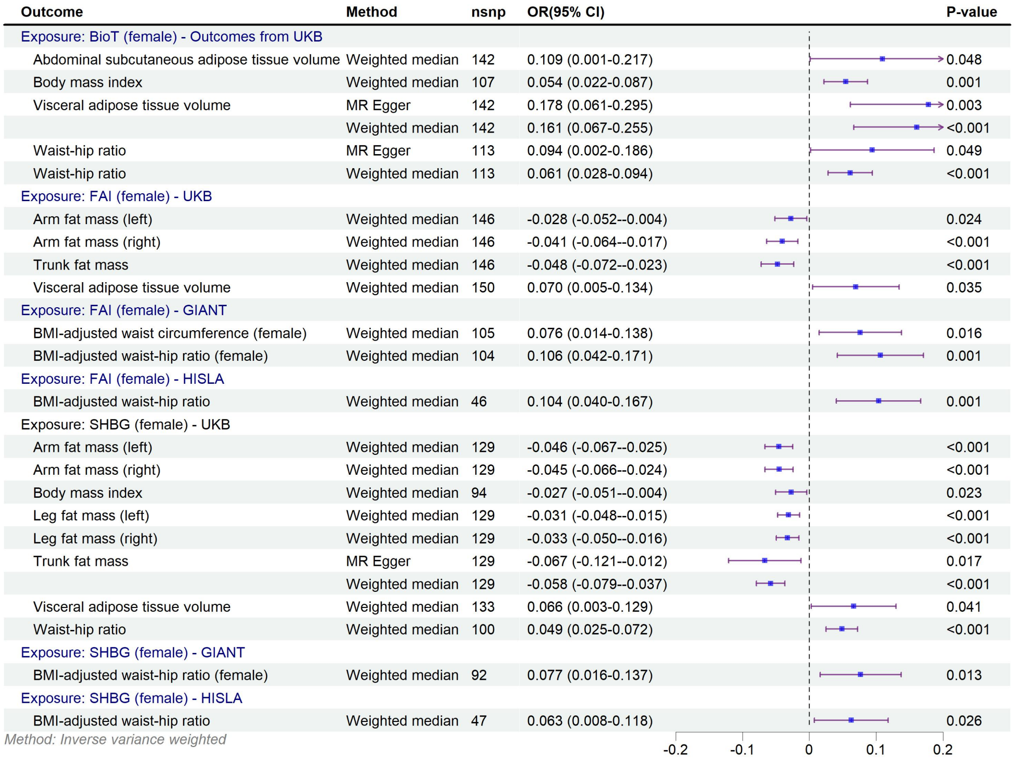

Figure S11. Forest plot of the Mendelian randomization study investigating the effect of sex hormones on obesity-related indicators in females.

Note: Abbreviations: BioT, bioavailable testosterone; BMI, body mass index; FAI, free androgen index; SHBG, single nucleotide polymorphisms. P-values less than 0.05 ($p < 0.05$) were considered significant.

Outcome	Method	nsnp	OR(95% CI)	P-value
Exposure: BMI-UKB				
BioT (female)	Inverse variance weighted	1165	0.245 (0.225-0.265)	<0.001
	MR Egger	1165	0.212 (0.165-0.258)	<0.001
	Weighted median	1165	0.222 (0.199-0.245)	<0.001
Estradiol (female)	MR Egger	991	-0.167 (-0.317--0.016)	0.031
FAI (female)	Inverse variance weighted	1165	-0.054 (-0.083--0.026)	<0.001
	MR Egger	1165	-0.086 (-0.153--0.019)	0.012
	Weighted median	1165	-0.086 (-0.118--0.054)	<0.001
Exposure: Leg fat mass (left)-UKB				
BioT (female)	Inverse variance weighted	355	0.271 (0.234-0.308)	<0.001
	MR Egger	355	0.180 (0.077-0.284)	<0.001
	Weighted median	355	0.269 (0.231-0.307)	<0.001
Exposure: Trunk fat mass-UKB				
BioT (female)	Inverse variance weighted	362	0.167 (0.136-0.198)	<0.001
	MR Egger	362	0.102 (0.014-0.189)	0.023
	Weighted median	362	0.172 (0.140-0.205)	<0.001
FAI (female)	Inverse variance weighted	362	-0.111 (-0.155--0.068)	<0.001
	MR Egger	362	-0.217 (-0.338--0.096)	<0.001
	Weighted median	362	-0.080 (-0.121--0.039)	<0.001
Exposure: Visceral adipose tissue volume-UKB				
BioT (female)	Weighted median	5	-0.091 (-0.177--0.004)	0.039
FAI (female)	Inverse variance weighted	5	-0.275 (-0.467--0.083)	0.005
	Weighted median	5	-0.199 (-0.324--0.074)	0.002
Exposure: Waist circumference-UKB				
BioT (female)	Inverse variance weighted	38	0.165 (0.091-0.239)	<0.001
	Weighted median	38	0.145 (0.088-0.201)	<0.001
Estradiol (female)	Inverse variance weighted	31	-0.254 (-0.426--0.082)	0.004
	Weighted median	31	-0.316 (-0.565--0.068)	0.013
FAI (female)	Inverse variance weighted	38	-0.135 (-0.247--0.022)	0.02
	Weighted median	38	-0.114 (-0.199--0.029)	0.009
SHBG (female)	Inverse variance weighted	17	4.415 (2.028-6.802)	<0.001
	Weighted median	17	0.372 (0.142-0.603)	0.002
Exposure: Waist-hip ratiol-UKB				
Estradiol (female)	Inverse variance weighted	239	-0.102 (-0.190--0.013)	0.025
FAI (female)	Inverse variance weighted	286	0.263 (0.189-0.337)	<0.001
	Weighted median	286	0.237 (0.191-0.283)	<0.001
SHBG (female)	Inverse variance weighted	156	1.615 (0.152-3.078)	0.03
Exposure: BMI (female)-GIANT				
BioT (female)	Inverse variance weighted	35	0.120 (0.089-0.151)	<0.001
	Weighted median	35	0.101 (0.060-0.142)	<0.001
FAI (female)	Inverse variance weighted	35	-0.143 (-0.198--0.088)	<0.001
	MR Egger	35	-0.271 (-0.414--0.129)	<0.001
	Weighted median	35	-0.146 (-0.205--0.087)	<0.001
Exposure: BMI-adjusted waist circumference (female)-GIANT				
BioT (female)	Weighted median	22	0.087 (0.030-0.144)	0.003
FAI (female)	Inverse variance weighted	22	0.179 (0.047-0.310)	0.008
	Weighted median	22	0.144 (0.060-0.229)	<0.001
Exposure: BMI-adjusted waist-hip ratio (female)-GIANT				
BioT (female)	Inverse variance weighted	31	0.097 (0.056-0.138)	<0.001
	Weighted median	31	0.068 (0.023-0.113)	0.003
FAI (female)	Inverse variance weighted	31	0.259 (0.186-0.333)	<0.001
	MR Egger	31	0.359 (0.071-0.647)	0.021
	Weighted median	31	0.246 (0.175-0.316)	<0.001
SHBG (female)	Inverse variance weighted	19	-3.702 (-6.828--0.576)	0.02
Exposure: BMI-adjusted waist-hip ratio (female)-HISLA				
BioT (female)	Inverse variance weighted	48	0.089 (0.023-0.155)	0.008
	Weighted median	48	0.094 (0.036-0.152)	0.001
Estradiol (female)	Weighted median	37	-0.252 (-0.496--0.009)	0.042
FAI (female)	Inverse variance weighted	48	0.246 (0.134-0.359)	<0.001
	Weighted median	48	0.260 (0.178-0.343)	<0.001
Exposure: BMI (female)-HISLA				
BioT (female)	Inverse variance weighted	106	0.149 (0.107-0.192)	<0.001
	MR Egger	106	0.140 (0.018-0.261)	0.027
	Weighted median	106	0.154 (0.113-0.196)	<0.001
Estradiol (female)	Inverse variance weighted	87	-0.183 (-0.320--0.047)	0.008
	Weighted median	87	-0.208 (-0.391--0.024)	0.026
FAI (female)	Inverse variance weighted	106	-0.136 (-0.195--0.076)	<0.001
	Weighted median	106	-0.105 (-0.162--0.049)	<0.001
SHBG (female)	Inverse variance weighted	64	2.580 (0.060-5.100)	0.045

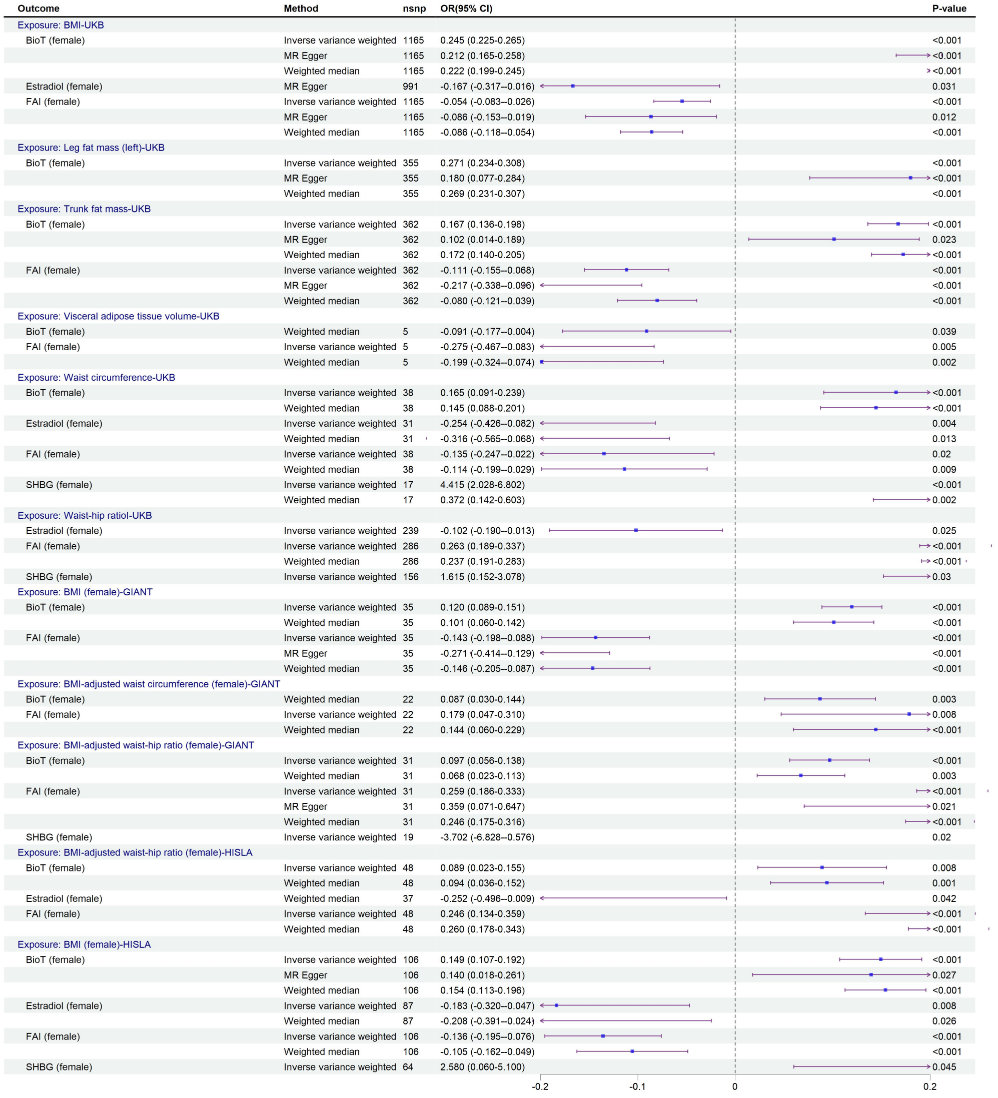

Figure S12. Forest plot of the Mendelian randomization study investigating the effect of obesity-related indicators on sex hormones in females.

Note: Abbreviations: BioT, bioavailable testosterone; BMI, body mass index; FAI, free androgen index; SHBG, single nucleotide polymorphisms. P-values less than 0.05 (p< 0.05) were considered significant.

Outcome	Method	nsnp	OR(95% CI)	P-value
Exposure: Arm fat mass (left)-UKB				
BioT (male)	Inverse variance weighted	355	-0.175 (-0.206--0.144)	<0.001
	MR Egger	355	-0.237 (-0.322--0.153)	<0.001
	Weighted median	355	-0.177 (-0.214--0.140)	<0.001
Exposure: Arm fat mass (left)-UKB				
Estradiol (male)	Inverse variance weighted	355	0.018 (0.011-0.024)	<0.001
	MR Egger	355	0.019 (0.001-0.036)	0.039
	Weighted median	355	0.015 (0.005-0.025)	0.003
Exposure: Arm fat mass (right)-UKB				
BioT (male)	Inverse variance weighted	365	-0.173 (-0.205--0.142)	<0.001
	MR Egger	365	-0.230 (-0.315--0.145)	<0.001
	Weighted median	365	-0.181 (-0.216--0.145)	<0.001
Exposure: Arm fat mass (right)-UKB				
Estradiol (male)	Inverse variance weighted	365	0.020 (0.014-0.026)	<0.001
	Weighted median	365	0.016 (0.007-0.025)	<0.001
Exposure: BMI-UKB				
BioT (male)	Inverse variance weighted	1165	-0.135 (-0.156--0.114)	<0.001
	MR Egger	1165	-0.153 (-0.202--0.103)	<0.001
	Weighted median	1165	-0.144 (-0.172--0.116)	<0.001
Exposure: BMI-UKB				
Estradiol (male)	Inverse variance weighted	1165	0.014 (0.009-0.018)	<0.001
	MR Egger	1165	0.019 (0.008-0.030)	<0.001
	Weighted median	1165	0.012 (0.003-0.020)	0.007
Exposure: BMI-UKB				
FAI (male)	Inverse variance weighted	1165	-0.051 (-0.082--0.021)	0.001
	Weighted median	1165	-0.085 (-0.117--0.053)	<0.001
Exposure: Leg fat mass (left)-UKB				
BioT (male)	Inverse variance weighted	355	-0.203 (-0.242--0.163)	<0.001
	MR Egger	355	-0.251 (-0.362--0.140)	<0.001
	Weighted median	355	-0.221 (-0.266--0.176)	<0.001
Exposure: Leg fat mass (left)-UKB				
Estradiol (male)	Inverse variance weighted	355	0.021 (0.013-0.029)	<0.001
	Weighted median	355	0.017 (0.004-0.030)	0.01
Exposure: Leg fat mass (left)-UKB				
FAI (male)	Inverse variance weighted	355	-0.106 (-0.161--0.051)	<0.001
	MR Egger	355	-0.195 (-0.349--0.041)	0.013
	Weighted median	355	-0.132 (-0.185--0.078)	<0.001
Exposure: Leg fat mass (right)-UKB				
BioT (male)	Inverse variance weighted	346	-0.188 (-0.228--0.149)	<0.001
	MR Egger	346	-0.269 (-0.379--0.159)	<0.001
	Weighted median	346	-0.215 (-0.261--0.169)	<0.001
Exposure: Leg fat mass (right)-UKB				
Estradiol (male)	Inverse variance weighted	346	0.020 (0.012-0.028)	<0.001
Exposure: Leg fat mass (right)-UKB				
FAI (male)	Inverse variance weighted	346	-0.083 (-0.133--0.032)	0.001
	MR Egger	346	-0.203 (-0.344--0.062)	0.005
	Weighted median	346	-0.116 (-0.168--0.064)	<0.001
Exposure: Trunk fat mass-UKB				
BioT (male)	Inverse variance weighted	362	-0.179 (-0.211--0.147)	<0.001
	MR Egger	362	-0.226 (-0.315--0.137)	<0.001
	Weighted median	362	-0.166 (-0.202--0.131)	<0.001
Exposure: Trunk fat mass-UKB				
Estradiol (male)	Inverse variance weighted	362	0.015 (0.008-0.021)	<0.001
	Weighted median	362	0.014 (0.005-0.023)	0.004
Exposure: Trunk fat mass-UKB				
FAI (male)	Inverse variance weighted	362	-0.119 (-0.160--0.077)	<0.001
	MR Egger	362	-0.190 (-0.307--0.073)	0.002
	Weighted median	362	-0.119 (-0.160--0.078)	<0.001
Exposure: Waist circumference-UKB				
BioT (male)	Inverse variance weighted	38	-0.121 (-0.214--0.027)	0.012
Exposure: Waist circumference-UKB				
Estradiol (male)	Inverse variance weighted	38	0.014 (0.003-0.025)	0.012
Exposure: Waist-hip ratio-UKB				
Estradiol (male)	Inverse variance weighted	286	-0.016 (-0.026--0.006)	0.002
Exposure: Waist-hip ratio-UKB				
FAI (male)	Inverse variance weighted	286	0.166 (0.079-0.253)	<0.001
	Weighted median	286	0.096 (0.052-0.140)	<0.001
Exposure: BMI (male)-GIANT				
BioT (male)	Inverse variance weighted	28	-0.160 (-0.239--0.081)	<0.001
	Weighted median	28	-0.171 (-0.229--0.112)	<0.001
Estradiol (male)	Inverse variance weighted	28	0.014 (0.005-0.024)	0.003
FAI (male)	Inverse variance weighted	28	-0.116 (-0.219--0.012)	0.029
	Weighted median	28	-0.105 (-0.173--0.037)	0.002
Exposure: BMI-HISLA				
BioT (male)	Inverse variance weighted	106	-0.135 (-0.188--0.082)	<0.001
	MR Egger	106	-0.178 (-0.331--0.025)	0.025
	Weighted median	106	-0.122 (-0.173--0.071)	<0.001
Estradiol (male)	Inverse variance weighted	106	0.011 (0.003-0.019)	0.007
FAI (male)	Inverse variance weighted	106	-0.117 (-0.186--0.048)	<0.001
Exposure: BMI-adjusted waist-hip ratio-HISLA				
FAI (male)	Inverse variance weighted	48	0.098 (0.013-0.183)	0.024
	Weighted median	48	0.076 (0.004-0.149)	0.039

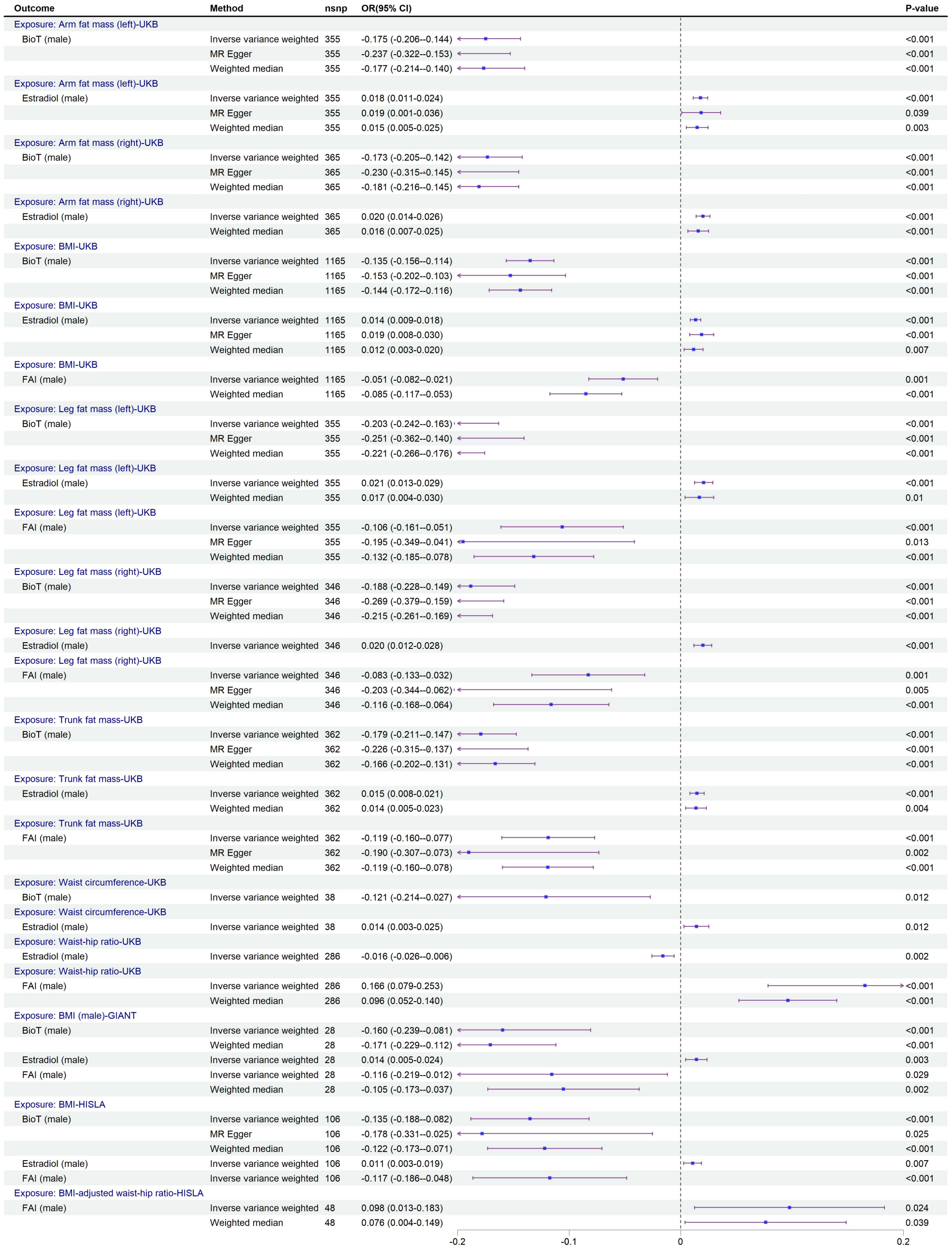

Figure S13. Forest plot of the Mendelian randomization study investigating the effect of obesity-related indicators on sex hormones in males.

Note: Abbreviations: BioT, bioavailable testosterone; BMI, body mass index; FAI, free androgen index; SHBG, single nucleotide polymorphisms. P-values less than 0.05 ($p < 0.05$) were considered significant.

Table S1. Detailed data of GWAS studies included in the MR analyses.

Properties	Sample size	SNP	Ancestry	GWAS ID	Data source
**FAI (female)**	158,208	17,239,131	European	GCST90239824	https://www.ebi.ac.uk/gwas/studies/GCST90239824
**FAI (male)**	162,068	17,239,131	European	GCST90239823	https://www.ebi.ac.uk/gwas/studies/GCST90239823
**BioT (female)**	188,507	16,585,744	European	GCST90012102	https://www.ebi.ac.uk/gwas/studies/GCST90012102
**BioT (male)**	178782	16,577,423	European	GCST90012103	https://www.ebi.ac.uk/gwas/studies/GCST90012103
**Estradiol (female)**	37,461	7,870,546	European	GCST90020092	https://www.ebi.ac.uk/gwas/studies/GCST90020092
**Estradiol (male)**	206,927	16,582,267	European	GCST90012105	https://www.ebi.ac.uk/gwas/studies/GCST90012105
**SHBG (female)**	214,989	12,321,875	European	ieu-b-4870	https://gwas.mrcieu.ac.uk/datasets/ieu-b-4870/
**SHBG (male)**	185,221	12,321,875	European	ieu-b-4871	https://gwas.mrcieu.ac.uk/datasets/ieu-b-4871/
**BMI**	694,649	6,206,408	European	GCST90179150	https://www.ebi.ac.uk/gwas/studies/GCST90179150
**Waist circumference**	232,101	2,565,408	European	ieu-a-61	https://gwas.mrcieu.ac.uk/datasets/ieu-a-61/
**Waist-hip ratio**	502,773	12,007,603	European	GCST90029009	https://www.ebi.ac.uk/gwas/studies/GCST90029009
**Leg fat mass (right)**	454,846	9,851,867	European	ukb-b-18096	https://gwas.mrcieu.ac.uk/datasets/ukb-b-18096/
**Leg fat mass (left)**	454,823	9,851,867	European	ukb-b-7212	https://gwas.mrcieu.ac.uk/datasets/ukb-b-7212/

**Arm fat mass (right)**	454,757	9,851,867	European	ukb-b-6704	https://gwas.mrcieu.ac.uk/datasets/ukb-b-6704/
**Arm fat mass (left)**	454,684	9,851,867	European	ukb-b-8338	https://gwas.mrcieu.ac.uk/datasets/ukb-b-8338/
**Trunk fat mass**	454,588	9,851,867	European	ukb-b-20044	https://gwas.mrcieu.ac.uk/datasets/ukb-b-20044/
**Abdominal subcutaneous adipose tissue volume**	32,860	9,275,407	European	ebi-a-GCST90016672	https://gwas.mrcieu.ac.uk/datasets/ebi-a-GCST90016672/
**Visceral adipose tissue volume**	32,860	9,275,407	European	ebi-a-GCST90016671	https://gwas.mrcieu.ac.uk/datasets/ebi-a-GCST90016671/
**BMI**	330,793	2,427,043	American	GCST90095039	https://www.ebi.ac.uk/gwas/studies/GCST90095039
**BMI-adjusted WHR**	205,581	2,401,745	European	GCST90095040	https://www.ebi.ac.uk/gwas/studies/GCST90095040
**BMI (female)**	171,977	2,494,613	European	ieu-a-974	https://gwas.mrcieu.ac.uk/datasets/ieu-a-974/
**BMI (male)**	152,893	2,477,659	European	ieu-a-785	https://gwas.mrcieu.ac.uk/datasets/ieu-a-785/
**BMI-adjusted WC**	127,469	2,473,036	European	ieu-a-69	https://gwas.mrcieu.ac.uk/datasets/ieu-a-69/
**BMI-adjusted WC**	104,079	2,294,966	European	ieu-a-71	https://gwas.mrcieu.ac.uk/datasets/ieu-a-71/
**BMI-adjusted HC**	117,339	2,466,815	European	ieu-a-57	https://gwas.mrcieu.ac.uk/datasets/ieu-a-57/
**BMI-adjusted HC**	93,963	2,188,856	European	ieu-a-59	https://gwas.mrcieu.ac.uk/datasets/ieu-a-59/

**BMI-adjusted WHR**	116,740	2,467,779	European	ieu-a-81	https://gwas.mrcieu.ac.uk/datasets/ieu-a-81/
**BMI-adjusted WHR**	93,478	2,146,137	European	ieu-a-83	https://gwas.mrcieu.ac.uk/datasets/ieu-a-83/

Note: Abbreviations: BioT, bioavailable testosterone; BMI, body mass index; FAI, free androgen index; N, number of participants; SHBG, single nucleotide polymorphisms; HC, hip circumference; WC, waist circumference; WHR, waist-hip ratio.

Table S2. Association of estradiol with 16 obesity-related indicators in males from NHANES 2013-2016 (n=2870).

		Men- Estradiol, pg/mL								
		Q1(Low)			Q2(Medium)			Q3(High)		
		β (95% CI)	standardized β	P-value	β (95% CI)	standardized β	P-value	β (95% CI)	standardized β	P-value
**Body Measures**										
Body Mass Index, kg/m^2	Model 1	0.206(0.096,0.316)	0.137	<0.001	0.236(0.055,0.418)	0.082	0.012	0.056(-0.019,0.131)	0.072	0.140
	Model 2	0.161(0.063,0.259)	0.107	0.002	0.233(0.049,0.418)	0.081	0.015	0.073(0.004,0.141)	0.094	0.038
Waist Circumference, cm	Model 1	0.530(0.235,0.823)	0.125	<0.001	0.456(-0.046,0.958)	0.058	0.074	0.116(-0.073,0.304)	0.057	0.220
	Model 2	0.388(0.125,0.650)	0.092	0.005	0.446(-0.056,0.949)	0.057	0.080	0.157(-0.012,0.326)	0.077	0.068
Arm Circumference, cm	Model 1	0.294(0.169,0.418)	0.222	<0.001	0.171(0.035,0.307)	0.075	0.016	0.026(-0.024,0.075)	0.044	0.298
	Model 2	0.227(0.116,0.338)	0.172	<0.001	0.173(0.032,0.313)	0.076	0.018	0.033(-0.012,0.077)	0.056	0.147
Average Sagittal Abdominal	Model 1	0.120(0.046,0.194)	0.107	0.003	0.137(0.002,0.272)	0.066	0.046	0.028(-0.022,0.078)	0.053	0.259

Diameter, cm	Model 2	0.081(0.016,0.146)	0.072	0.016	0.131(-0.006,0.267)	0.063	0.060	0.040(-0.006,0.086)	0.075	0.084
**Android/ Gynoid Measurements**										
Android fat mass, gram	Model 1	30.376(13.430,47.323)	0.101	0.001	44.530(0.416,88.643)	0.070	0.048	15.014(-1.506,31.533)	0.090	0.073
	Model 2	21.324(6.723,35.925)	0.071	0.006	42,197(-1.828,86.222)	0.067	0.060	19.701(4.800,34.601)	0.118	0.011
Gynoid fat mass, gram	Model 1	52.210(19.173,85.247)	0.141	0.003	52.521(6.633,98.409)	0.066	0.026	17.317(-0.807,35.440)	0.083	0.060
	Model 2	39.783(10.223,69.343)	0.107	0.010	52.324(6.405,98.244)	0.066	0.027	22.473(5.422,39.523)	0.108	0.012
Subcutaneous fat mass, gram	Model 1	13.507(1.866,25.149)	0.077	0.024	24.225(0.664,47.786)	0.070	0.044	7.143(-1.832,16.117)	0.077	0.115
	Model 2	8.040(-2.258,18.339)	0.046	0.121	23.593(0.051,47.134)	0.068	0.050	9.559(1.468,17.650)	0.103	0.022
Total abdominal fat mass, gram	Model 1	15.884(2.174,29.595)	0.070	0.025	30.588(-0.191,61.367)	0.069	0.051	8.034(-3.049,19.116)	0.070	0.149
	Model 2	9.926(-1.828,21.681)	0.044	0.095	29.250(-1.251,59.751)	0.066	0.060	11.210(1.066,21.354)	0.097	0.032
Visceral adipose tissue mass, gram	Model 1	2.377(-1.196,5.950)	0.035	0.184	6.363(-3.239,15.966)	0.050	0.186	0.891(-1.745,3.529)	0.029	0.496
	Model 2	1.886(-1.481,5.253)	0.027	0.262	5.658(-3.694,15.009)	0.044	0.226	1.651(-0.959,4.261)	0.053	0.206

**Whole Body measurements**										
Head Fat, gram	Model 1	8.604(5.916,11.293)	0.218	<0.001	3.564(-1.725,8.853)	0.050	0.179	0.505(-1.101,2.111)	0.027	0.525
	Model 2	7.293(4.762,9.824)	0.185	<0.001	3.628(-1.802,9.057)	0.051	0.183	1.012(-0.563,2.587)	0.054	0.199
Left Arm Fat, gram	Model 1	11.774(-0.979，24.526)	0.074	0.069	16.954(-4.315,32.223)	0.052	0.114	8.549(-0.446,17.544)	0.090	0.062
	Model 2	8.723(-2.938,20.383)	0.055	0.137	17.433(-3.627,38.492)	0.054	0.101	10.464(1.901,19.028)	0.110	0.018
Left Leg Fat, gram	Model 1	26.633(-13.969,67.235)	0.063	0.190	48.751(-1.007,98.509)	0.057	0.055	22.798(1.469,44.126)	0.098	0.037
	Model 2	20.569(-17.546,58.684)	0.049	0.279	50.847(4.576,97.119)	0.060	0.032	27.843(7.182,48.503)	0.120	0.010
Right Arm Fat, gram	Model 1	10.982(-2.440,24.404)	0.068	0.105	15.619(-6.331,37.568)	0.048	0.157	8.684(-0.685,18.053)	0.091	0.068
	Model 2	8.402(-3.528,20.332)	0.052	0.161	15.646(-6.217,37.509)	0.048	0.154	10.709(1.853,19.565)	0.112	0.019
Right Leg Fat, gram	Model 1	26.028(-15.072,67.128)	0.061	0.206	46.992(-2.847,96.830)	0.054	0.064	22.460(1.214,43.706)	0.095	0.039
	Model 2	20.192(-18.407,58.792)	0.047	0.294	48.943(1.912,95.975)	0.057	0.042	28.013(7.472,48.554)	0.119	0.009
Trunk Fat, gram	Model 1	137.014(50.328,223.701)	0.098	0.003	213.491(5.844,421.139)	0.074	0.044	74.592(0.279,148.906)	0.096	0.049

	Model 2	93.999(21.474,166.524)	0.067	0.013	202.544(-7.125,412.213)	0.070	0.058	97.613(29.519,165.707)	0.126	0.007
Total Fat, gram	Model 1	221.034(35.954,406.114)	0.090	0.021	345.370(5.071,685.670)	0.068	0.047	137.588(5.791,269.385)	0.099	0.041
	Model 2	159.177(-0.872,319.226)	0.065	0.051	339.041(3.577,674.505)	0.067	0.048	175.654(52.852,298.456)	0.126	0.007

Note: Model 1 was adjusted for age and ethnicity. Model 2 was adjusted for age, ethnicity, education level, poverty status, smoking status, HEI-2015, alcohol consumption and leisure-time physical activity. Abbreviations: CI, confidence interval; P-values less than 0.05 (P-value < 0.05) were considered significant.

Table S3. Association of SHBG with 16 obesity-related indicators in males from NHANES 2013-2016 (n=2870).

		Men- SHBG, nmol/L								
		Q1(Low)			Q2(Medium)			Q3(High)		
		β (95% CI)	standardized β	P-value	β (95% CI)	standardized β	P-value	β (95% CI)	standardized β	P-value
**Body Measures**										
Body Mass Index, kg/m^2	Model 1	-0.305(-0.434,-0.177)	-0.225	<0.001	-0.185(-0.291,-0.079)	-0.116	0.001	-0.065(-0.084,-0.045)	-0.219	<0.001
	Model 2	-0.280(-0.406,-0.155)	-0.206	<0.001	-0.166(-0.272,-0.059)	-0.104	0.004	-0.060(-0.080,-0.039)	-0.202	<0.001
Waist Circumference, cm	Model 1	-0.863(-1.159,-0.568)	-0.248	<0.001	-0.496(-0.796,-0.197)	-0.113	0.002	-0.172(-0.223,-0.121)	-0.199	<0.001
	Model 2	-0.789(-1.075,-0.523)	-0.227	<0.001	-0.437(-0.721,-0.153)	-0.100	0.004	-0.161(-0.215,-0.107)	-0.184	<0.001
Arm Circumference, cm	Model 1	-0.218(-0.310,-0.126)	-0.211	<0.001	-0.145(-0.223,-0.067)	-0.111	<0.001	-0.064(-0.084,-0.044)	-0.247	<0.001
	Model 2	-0.201(-0.296,-0.107)	-0.195	<0.001	-0.130(-0.211,-0.049)	-0.100	0.003	-0.057(-0.076,-0.038)	-0.220	<0.001
Average Sagittal Abdominal	Model 1	-0.217(-0.296,-0.137)	-0.236	<0.001	-0.133(-0.209,-0.057)	-0.114	0.001	-0.044(-0.059,-0.030)	-0.195	<0.001

Diameter, cm	Model 2	-0.195(-0.269,-0.121)	-0.212	<0.001	-0.118(-0.192,-0.144)	-0.101	0.003	-0.042(-0.058,-0.025)	-0.186	<0.001
**Android/ Gynoid Measurements**										
Android fat mass, gram	Model 1	-67.467(-93.394,-41.541)	-0.233	<0.001	-42.207(-65.639,-18.775)	-0.124	<0.001	-12.688(-16.810,-8.566)	-0.203	<0.001
	Model 2	-59.801(-84.632,-34.969)	-0.206	<0.001	-36.427(-59.845,-13.009)	-0.107	0.003	-11.785(-16.308,-7.262)	-0.189	<0.001
Gynoid fat mass, gram	Model 1	-74.175(-108.669,-39.682)	-0.201	<0.001	-48.077(-79.030,-17.124)	-0.115	0.004	-16.055(-21.669,-10.422)	-0.216	<0.001
	Model 2	-66.148(-99.129,-33.168)	-0.180	<0.001	-40.977(-73.127,-8.827)	-0.098	0.014	-14.182(-19.718,-8.647)	-0.190	<0.001
Subcutaneous fat mass, gram	Model 1	-37.211(-50.776,-23.646)	-0.235	<0.001	-25.454(-38.610,-12.298)	-0.133	<0.001	-7.354(-9.677,-5.032)	-0.215	<0.001
	Model 2	-33.258(-46.362,-20.154)	-0.210	<0.001	-22.224(-35.370,-9.078)	-0.116	0.002	-6.698(-9.141,-4.255)	-0.196	<0.001
Total abdominal fat mass, gram	Model 1	-47.139(-63.880,-30.398)	-0.243	<0.001	-29.433(-46.772,-12.095)	-0.121	0.002	-9.608(-12.503,-6.713)	-0.209	<0.001
	Model 2	-42.208(-58.239,-26.177)	-0.218	<0.001	-25.330(-42.366,-8.294)	-0.104	0.005	-9.028(-12.228,-5.828)	-0.197	<0.001
Visceral adipose tissue mass, gram	Model 1	-9.928(-14.016,-5.840)	-0.182	<0.001	-3.979(-9.001,1.042)	-0.055	0.116	-2.254(-3.149,-1.358)	-0.160	<0.001
	Model 2	-8.950(-12.946,-4.953)	-0.164	<0.001	-3.106(-7.942,1.731)	-0.043	0.200	-2.330(-3.302,-1.357)	-0.166	<0.001

**Whole Body measurements**										
Head Fat, gram	Model 1	-7.801(-11.105,-4.497)	-0.229	<0.001	-2.996(-7.254,1.263)	-0.075	0.161	-1.851(-2.333,-1.369)	-0.249	<0.001
	Model 2	-7.035(-10.177,-3.893)	-0.207	<0.001	-2.770(-6.892,1.351)	-0.069	0.180	-1.642(-2.140,-1.143)	-0.221	<0.001
Left Arm Fat, gram	Model 1	-36.067(-52.122,-20.012)	-0.213	<0.001	-19.931(-33.722,-6.140)	-0.111	0.006	-5.814(-8.119,-3.509)	-0.186	<0.001
	Model 2	-32.938(-48.789,-17.089)	-0.195	<0.001	-17.217(-31.072,-3.362)	-0.096	0.017	-5.468(-7.969,-2.966)	-0.175	<0.001
Left Leg Fat, gram	Model 1	-83.484(-122.089,-44.878)	-0.197	<0.001	-41.939(-75.796,-8.082)	-0.090	0.017	-14.601(-20.510,-8.691)	-0.188	<0.001
	Model 2	-76.759(-113.534,-39.983)	-0.182	<0.001	-35.589(-70.483,-0.695)	-0.076	0.046	-13.082(-19.001,-7.164)	-0.168	<0.001
Right Arm Fat, gram	Model 1	-35.377(-52.141,-18.613)	-0.208	<0.001	-22.062(-35.349,-8.766)	-0.121	0.002	-5.765(-8.061,-3.470)	-0.183	<0.001
	Model 2	-32.243(-48.794,-15.691)	-0.189	<0.001	-19.575(-32.956,-6.194)	-0.108	0.006	-5.436(-7.877,-2.995)	-0.173	<0.001
Right Leg Fat, gram	Model 1	-83.941(-122.141,-45.742)	-0.196	<0.001	-45.502(-80.817,-10.187)	-0.096	0.013	-14.890(-20.813,-8.967)	-0.187	<0.001
	Model 2	-77.156(-113.697,-40.614)	-0.180	<0.001	-38.559(-74.519,-2.599)	-0.081	0.036	-13.378(-19.406,-7.350)	-0.168	<0.001
Trunk Fat, gram	Model 1	-332.249(-450.266,-214.231)	-0.248	<0.001	-199.555(-302.336,-96.774)	-0.128	<0.001	-59.718(-79.149,-40.287)	-0.206	<0.001

	Model 2	-298.801(-411.180,-186.423)	-0.223	<0.001	-172.714(-276.265,-69.162)	-0.111	0.002	-54.607(-75.883,-33.332)	-0.188	<0.001
Total Fat, gram	Model 1	-578.919(-800,833,-357.004)	-0.238	<0.001	-331.986(-526.774,-137.197)	-0.121	0.002	-102.639(-137.788,-67.491)	-0.208	<0.001
	Model 2	-524.932(-737.285,-312.579)	-0.216	<0.001	-286.424(-483.878,-88.970)	-0.105	0.006	-93.613(-131.046,-56.197)	-0.190	<0.001

Note: Model 1 was adjusted for age and ethnicity. Model 2 was adjusted for age, ethnicity, education level, poverty status, smoking status, HEI-2015, alcohol consumption and leisure-time physical activity. Abbreviations: CI, confidence interval; SHBG, Sex hormone-binding globulin; P-values less than 0.05 (P-value < 0.05) were considered significant.

Table S4. Association of estradiol with 16 obesity-related indicators in females from NHANES 2013-2016 (n=2309).

		Female- Total Testosterone, ng/dL								
		Q1(Low)			Q2(Medium)			Q3(High)		
		β (95% CI)	standardized β	P-value	β (95% CI)	standardized β	P-value	β (95% CI)	standardized β	P-value
**Body Measures**										
Body Mass Index, kg/m^2	Model 1	-0.059(-0.317,0.198)	-0.027	0.641	-0.091(-0.285,0.102)	-0.034	0.342	0.004(-0.005,0.014)	0.015	0.376
	Model 2	-0.028(-0.261,0.206)	-0.013	0.811	-0.101(-0.297,0.096)	-0.037	0.305	0.008(-0.001,0.017)	0.031	0.082
Waist Circumference, cm	Model 1	-0.424(-1.038,0.189)	-0.083	0.168	-0.143(-0.641,0.356)	-0.022	0.563	-0.012(-0.038,0.014)	-0.019	0.361
	Model 2	-0.370(-0.911,0.170)	-0.072	0.172	-0.178(-0.669,0.313)	-0.028	0.464	-0.003(-0.027,0.021)	-0.005	0.805
Arm Circumference, cm	Model 1	-0.036(-0.233,0.161)	-0.022	0.712	-0.030(-0.167,0.108)	-0.014	0.664	0.001(-0.005,0.007)	0.005	0.713
	Model 2	-0.019(-0.197,0.159)	-0.012	0.830	-0.041(-0.184,0.101)	-0.020	0.555	0.004(-0.002,0.010)	0.020	0.210
Average Sagittal Abdominal	Model 1	-0.131(-0.283,0.021)	-0.095	0.088	-0.087(-0.229,0.055)	-0.052	0.220	-0.002(-0.009,0.006)	-0.013	0.663

Diameter, cm	Model 2	-0.107(-0.240,0.026)	-0.078	0.109	-0.091(-0.230,0.048)	-0.054	0.190	0.001(-0.006,0.008)	0.006	0.829
**Android/ Gynoid Measurements**										
Android fat mass, gram	Model 1	-22.219(-69.931,25.494)	-0.058	0.349	-30.278(-73.591,13.034)	-0.062	0.164	0.479(-1.136,2.094)	0.010	0.550
	Model 2	-16.564(-59.088,25.961)	-0.043	0.433	-32.985(-75.341,9.371)	-0.067	0.122	1.211(-0.226,2.647)	0.026	0.096
Gynoid fat mass, gram	Model 1	6.959(-69.557,83.476)	-0.012	0.854	-8.022(-61.957,45.914)	-0.011	0.763	0.762(-1.151,3.037)	0.011	0.499
	Model 2	13.196(-56.169,82.562)	-0.024	0.700	-5.566(-61.294,50.161)	-0.008	0.840	1.750(-0.361,3.861)	0.025	0.101
Subcutaneous fat mass, gram	Model 1	-5.353(-34.713,24.007)	-0.023	0.712	-6.023(-29.646,17.600)	-0.020	0.606	0.724(-0.278,1.726)	0.024	0.150
	Model 2	-2.263(-28.607,24.082)	-0.010	0.862	-8.162(-32.181,15.858)	-0.027	0.493	1.200(0.237,2.163)	0.040	0.016
Total abdominal fat mass, gram	Model 1	-15.838（-51.322，19.646）	-0.053	0.369	-16.659(-46.754,13.436)	-0.044	0.267	0.303(-0.837,1.442)	0.008	0.592
	Model 2	-11.638(-43.176,19.900)	-0.039	0.457	-18.408(-48.373,11.557)	-0.049	0.219	0.931(-0.107,1.970)	0.025	0.077
Visceral adipose tissue mass, gram	Model 1	-10.485(-18.137,-2.833)	-0.120	0.009	-10.636(-19.261,-2.010)	-0.107	0.017	-0.422(-0.994,0.151)	-0.050	0.143
	Model 2	-9.375(-16.371,-2.378)	-0.107	0.010	-10.247(-18.719,-1.774)	-0.103	0.019	0.303(-0.837,1.442)	0.036	0.592

**Whole Body measurements**										
Head Fat, gram	Model 1	-0.106(-3.757,3.545)	-0.003	0.953	-2.543(-6.209,1.124)	-0.054	0.167	0.047(-0.226,0.320)	0.010	0.725
	Model 2	0.017(-3.509,3.542)	0.0004	0.992	-2.917(-6.689,0.855)	-0.062	0.125	0.095(-0.146,0.337)	0.020	0.427
Left Arm Fat, gram	Model 1	-13.505(-41.243,14.234)	-0.052	0.328	-10.499(-34.226,13.228)	-0.034	0.373	0.001(-0.997,0.998)	0.00003	0.999
	Model 2	-10.189(-34.719,14.341)	-0.039	0.403	-10.415(-35.341,14.511)	-0.033	0.400	0.449(-0.542,1.439)	0.015	0.363
Left Leg Fat, gram	Model 1	7.143(-71.153,85.439)	0.011	0.853	-0.024(-54.737,54.690)	-0.00003	0.999	0.789(-1.019,2.597)	0.010	0.380
	Model 2	13.726(-56.974,84.425)	0.021	0.695	6.133(-51.861,64.126)	0.008	0.831	1.978(0.267,3.690)	0.025	0.025
Right Arm Fat, gram	Model 1	-13.224(-41.277,14.829)	-0.051	0.343	-9.479(-32.036,13.078)	-0.030	0.398	-0.063(-1.052,-0.926)	-0.002	0.897
	Model 2	-9.455(-34.501,15.591)	-0.037	0.447	-9.451(-32.521,13.620)	-0.030	0.409	0.373(-0.558,1.304)	0.012	0.420
Right Leg Fat, gram	Model 1	7.878(-73.790,89.546)	0.012	0.845	1.633(-54.634,57.899)	0.001	0.953	1.013(-0.939,2.964)	0.013	0.298
	Model 2	14.893(-57.674,89.460)	0.023	0.686	8.638(-51.267,68.543)	0.004	0.770	2.127(0.284,3.970)	0.027	0.025
Trunk Fat, gram	Model 1	-101.049(-341.563,139.465)	-0.053	0.400	-101.462(-294.631,91.708)	-0.042	0.292	0.567(-8.082,9.216)	0.002	0.895

	Model 2	-72.839(-287.164,141.485)	-0.038	0.493	-107.238(-300.301,85.824)	-0.044	0.266	4.318(-3.519,12.154)	0.019	0.269
Total Fat, gram	Model 1	-112.861(-566.109,340.387)	-0.032	0.615	-122.371(-445.810,201.068)	-0.027	0.446	2.353(-10.411,15.118)	0.005	0.709
	Model 2	-63.847(-469.046,341.351)	-0.018	0.750	-115.248(-453.294,222.798)	-0.026	0.492	9.339(-2.211,20.890)	0.022	0.109

Note: Model 1 was adjusted for age and ethnicity. Model 2 was adjusted for age, ethnicity, education level, poverty status, smoking status, HEI-2015, alcohol consumption and leisure-time physical activity. Abbreviations: CI, confidence interval; P-values less than 0.05 (P-value < 0.05) were considered significant.

Table S5. Association of SHBG with 16 obesity-related indicators in females from NHANES 2013-2016 (n=2309).

		Female- SHBG, nmol/L								
		Q1(Low)			Q2(Medium)			Q3(High)		
		β (95% CI)	standardized β	P-value	β (95% CI)	standardized β	P-value	β (95% CI)	standardized β	P-value
**Body Measures**										
Body Mass Index, kg/m^2	Model 1	-0.230(-0.279,-0.181)	-0.259	<0.001	-0.140(-0.240,-0.076)	-0.175	<0.001	0.003(-0.006,0.013)	0.022	0.493
	Model 2	-0.237(-0.289,-0.185)	-0.267	<0.001	-0.140(-0.200,-0.080)	-0.175	<0.001	0.004(-0.005,0.012)	0.029	0.377
Waist Circumference, cm	Model 1	-0.554(-0.697,-0.410)	-0.267	<0.001	-0.280(-0.411,-0.148)	-0.148	<0.001	0.009(-0.017,0.035)	0.027	0.503
	Model 2	-0.565(-0.706,-0.423)	-0.273	<0.001	-0.283(-0.404,-0.163)	-0.149	<0.001	0.009(-0.013,0.031)	0.027	0.400
Arm Circumference, cm	Model 1	-0.152(-0.188,-0.117)	-0.237	<0.001	-0.107(-0.160,-0.053)	-0.169	<0.001	0.003(-0.005,0.010)	0.028	0.467
	Model 2	-0.159(-0.199,-0.120)	-0.248	<0.001	-0.111(-0.160,-0.161)	-0.175	<0.001	0.002(-0.004,0.009)	0.019	0.462
Average Sagittal Abdominal	Model 1	-0.148(-0.179,-0.116)	-0.274	<0.001	-0.085(-0.121,-0.050)	-0.169	<0.001	0.002(-0.004,0.008)	0.023	0.547

Diameter, cm	Model 2	-0.150(-0.181,-0.119)	-0.278	<0.001	-0.085(-0.118,-0.052)	-0.169	<0.001	0.003(-0.003,0.008)	0.036	0.318
**Android/ Gynoid Measurements**										
Android fat mass, gram	Model 1	-42.294(-56.025,-28.564)	-0.260	<0.001	-26.938(-38.341,-15.536）	-0.194	<0.001	0.711(-1.388,2.809)	0.029	0.495
	Model 2	-43.606(-57.065,-30.146)	-0.268	<0.001	-26.867(-37.513,-16.220)	-0.194	<0.001	0.781(-0.991,2.552)	0.032	0.375
Gynoid fat mass, gram	Model 1	-46.302(-65.572,-27.032)	-0.196	<0.001	-29.970(-45.328,-14.613)	-0.137	<0.001	1.730(-0.810,4.269)	0.048	0.174
	Model 2	-49.601(-68.731,-30.471)	-0.210	<0.001	-30.924(-44.826,-17.022）	-0.141	<0.001	1.429(-0.832,3.689)	0.039	0.207
Subcutaneous fat mass, gram	Model 1	-23.018(-30.561,-15.476)	-0.248	<0.001	-15.515(-22.001,-9.028)	-0.171	<0.001	0.713(-0.628,2.053)	0.046	0.286
	Model 2	-23.702(-31.282,-16.121)	-0.255	<0.001	-15.628(-21.670,-9.587)	-0.172	<0.001	0.711(-0.423,1.845)	0.046	0.210
Total abdominal fat mass, gram	Model 1	-29.324(-37.779，-20.870）	-0.250	<0.001	-20.399(-28.663,-12.145)	-0.181	<0.001	0.639(-1.034,2.312)	0.033	0.441
	Model 2	-30.065(-38.574,-21.557)	-0.256	<0.001	-20.302(-28.027,-12.578)	-0.180	<0.001	0.707(-0.689,2.103)	0.037	0.309
Visceral adipose tissue mass, gram	Model 1	-6.306(-8.432,-4.180)	-0.182	<0.001	-4.884(-7.303,-2.465)	-0.164	<0.001	-0.074(-0.457,0.310)	-0.016	0.698
	Model 2	-6.364(-8.433,-4.294)	-0.184	<0.001	-4.674(-6.966,-2.381)	-0.157	<0.001	-0.004(-0.325,0.317)	-0.001	0.980

**Whole Body measurements**										
Head Fat, gram	Model 1	-4.246(-5.458,-3.033)	-0.262	<0.001	-1.571(-2.877,-0.265)	-0.107	0.020	0.020(-0.162,0.201)	0.008	0.825
	Model 2	-4.321(-5.601,-3.040)	-0.267	<0.001	-1.566(-2.851,-0.280)	-0.163	0.019	0.002(-0.174,0.179)	0.001	0.980
Left Arm Fat, gram	Model 1	-26.687(-33.278,-20.096)	-0.257	<0.001	-14.805(-23.160,-6.450)	-0.159	0.001	0.691(-0.584,1.966)	0.042	0.277
	Model 2	-27.425(-34.736,-10.114)	-0.264	<0.001	-14.874(-22.594,-7.154)	-0.160	<0.001	0.749(-0.409,1.907)	0.046	0.196
Left Leg Fat, gram	Model 1	-46.449(-66.619,-26.280)	-0.175	<0.001	-33.842(-52.346,-15.337)	-0.135	<0.001	1.936(-0.767,4.639)	0.046	0.154
	Model 2	-49.891(-71.004,-28.779)	-0.188	<0.001	-34.422(-51.572,-17.273)	-0.137	<0.001	1.679(-0.736,4.093)	0.040	0.166
Right Arm Fat, gram	Model 1	-28.590(-35.259,-21.921)	-0.273	<0.001	-14.431(-21.978,-6.883)	-0.156	<0.001	0.749(-0.552,2.050)	0.046	0.249
	Model 2	-29.453(-36.724,-22.781)	-0.281	<0.001	-14.487(-21.363,-7.610)	-0.156	<0.001	0.821(-0.346,1.989)	0.050	0.161
Right Leg Fat, gram	Model 1	-45.207(-65.343,-25.071)	-0.167	<0.001	-33.606(-52.584,-14.628)	-0.131	0.001	1.881(-0.909,4.671)	0.044	0.179
	Model 2	-48.720(-69.799,-27.641)	-0.180	<0.001	-34.140(-51.697,-16.583)	-0.133	<0.001	1.568(-0.917,4.052)	0.036	0.207
Trunk Fat, gram	Model 1	-216.519(-278.593,-154.444)	-0.275	<0.001	-125.263(-181.090,-69.436)	-0.180	<0.001	4.679(-5.612,14.970)	0.039	0.361

	Model 2	-224.675(-287.901,-161.449)	-0.286	<0.001	-125.044(-176.346,-73.742)	-0.179	<0.001	4.993(-3.691,13.677)	0.041	0.250
Total Fat, gram	Model 1	-367.697(-473.738,-261.657)	-0.253	<0.001	-223.517(-318.780,-128.254)	-0.168	<0.001	9.956(-7.798,27.709)	0.043	0.261
	Model 2	-384.484(-495.651,-273.317)	-0.265	<0.001	-224.533(-310.818,-138.248)	-0.169	<0.001	9.812(-5.333,24.957)	0.042	0.196

Note: Model 1 was adjusted for age and ethnicity. Model 2 was adjusted for age, ethnicity, education level, poverty status, smoking status, HEI-2015, alcohol consumption and leisure-time physical activity. Abbreviations: CI, confidence interval; SHBG, Sex hormone-binding globulin; P-values less than 0.05 (P-value < 0.05) were considered.

Table S6. nSNP under different P-value thresholds.

Exposure	Threshold of P-value	nSNP
BioT (female)	5×10^{-8}	15,823
BioT (male)	5×10^{-8}	9470,746
Estradiol (female)	5×10^{-8}	8
	5×10^{-5}	1,177
Estradiol (male)	5×10^{-8}	3,702
	5×10^{-6}	6,037
FAI (female)	5×10^{-8}	9848,809
FAI (male)	5×10^{-8}	9842,515
SHBG (female)	5×10^{-8}	7,603
SHBG (male)	5×10^{-8}	12,020

Note: Abbreviations: BioT, bioavailable testosterone; FAI, free androgen index; SHBG, single nucleotide polymorphisms. P-values less than 0.05 ($p < 0.05$) were considered significant.
